# Traded Plastic, Traded Impacts? Designing Counterfactual Scenarios to Assess Environmental Impacts of Global Plastic Waste Trade

**DOI:** 10.1021/acs.est.4c02149

**Published:** 2024-05-10

**Authors:** Kai Li, Hauke Ward, Hai Xiang Lin, Arnold Tukker

**Affiliations:** † Institute of Environmental Sciences (CML), Leiden University, 2333 CC Leiden, The Netherlands; ‡ Delft Institute of Applied Mathematics, Delft University of Technology, 2628 CD Delft, The Netherlands; § Netherlands Organization for Applied Scientific Research TNO, 2595 DA The Hague, The Netherlands

**Keywords:** plastic waste import and export, plastic waste treatment, plastic footprint, environmental impact of trade, life cycle assessment, waste colonialism, plastic pollution, environmental justice

## Abstract

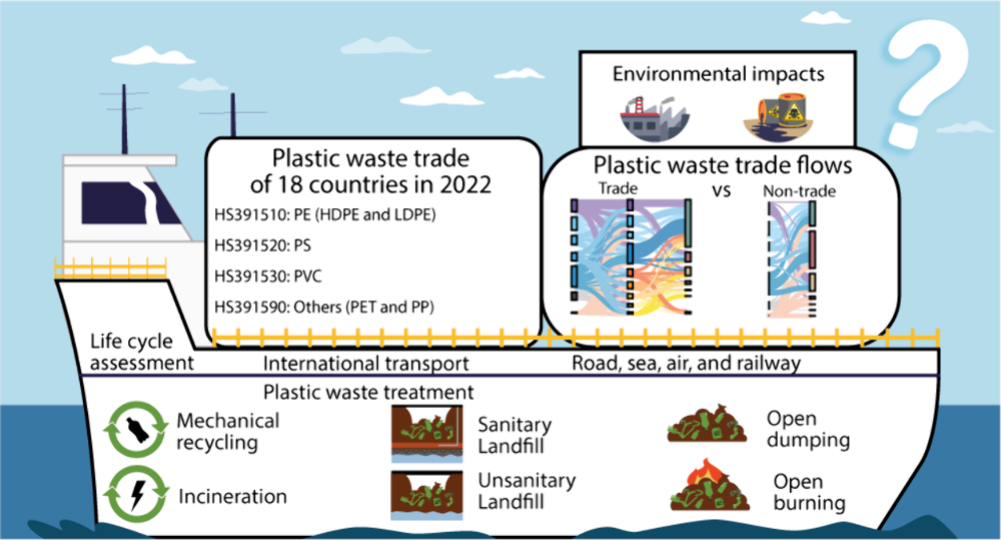

The global trade of plastic waste has raised environmental concerns, especially regarding pollution in waste-importing countries. However, the overall environmental contribution remains unclear due to uncertain treatment shares between handling plastic waste abroad and domestically. Here, we conduct a life cycle assessment of global plastic waste trade in 2022 across 18 countries and six plastic waste types, alongside three “nontrade” counterfactual scenarios. By considering the required cycling rate, which balances importers’ costs and recycling revenues, we find that the trade resulted in lower environmental impacts than treating domestically with the average treatment mix. The trade scenario alone reduced climate change impact by 2.85 million tonnes of CO_2_ equivalent and mitigated damages to ecosystem quality, human health, and resource availability by 12 species-years, 6200 disability-adjusted life years (DALYs), and 1.4 billion United States dollars (USD in 2013), respectively. These results underscore the significance of recognizing plastic waste trade as a pivotal factor in regulating global secondary plastic production when formulating a global plastics treaty.

## Abstract 2

This study examines the overall environmental impact of the plastic waste trade in 2022. The trade led to lower environmental impacts compared to treating domestically with the average treatment mix.

## 1 Introduction

Traded plastic waste has challenged waste management in importing countries.^1^ It causes multifaceted environmental issues that damage ecosystems, human health, and natural resources.^2^ Improper waste management in importing countries can degrade land and water quality, increase air pollution, or harm biodiversity and overall ecosystem health.^3^ A 2022 Greenpeace investigation reveals alarmingly high levels of toxic pollutants like dioxins, furans, and polychlorinated biphenyls in five Turkish dumpsites that received UK grocery packaging.^4^ Beyond these environmental impacts, the plastic waste trade underscores a significant ethical dilemma: the transfer of responsibility for waste treatment from wealthier to less affluent nations, with potentially lower capabilities. OECD member countries, for instance, have accounted for 87% of global plastic waste exports since reporting began in 1988.^5^ Recently, regulations aimed at reducing adverse environmental impacts have been implemented. Following China’s ban on plastic waste imports in 2017, most Southeast Asia countries tightened their national borders to curb the rerouted plastic waste streams.^6^ Moreover, international regulations have been strengthened, as evidenced by initiatives such as the EU Waste Shipment Regulation and the Basel Convention Plastic Amendments, both of which came into effect in 2021.^7,8^ Given this context, there is a pressing need to more accurately assess the environmental impact of the plastic waste trade to frame well-informed policies.

Several studies have assessed the environmental impact of plastic waste trade, particularly in light of China’s plastic import ban. Ren et al.^9^ indicate that the trade ban exacerbates environmental consequences since it leads to lower recycling rates, and hence higher virgin plastic production and associated carbon emissions. Their analysis shows that postban reductions in shipping and sorting are not sufficient for an offset, leading to a global increase of CO_2_ emissions of approximately 4.5 million tonnes per year (Mt y^–1^). However, the assessment has certain limitations such as the aggregation of Chinese trade partners and assuming uniform treatment practices across trade partners. Sun and Tabata^10^ similarly assert that the trade ban enhances net environmental impacts, due to a rise in virgin plastic production. Their findings show an increase in postban carbon emissions related to plastic consumption in both China and Japan. A life cycle assessment (LCA) by Wen et al.^1^ suggests as well that China’s ban has enhanced global carbon emissions, but led to a reduction of other impacts. Their approach, assuming that waste-importing countries treat the imported plastic waste with their domestic average plastic recycling rates, might underestimate the environmental benefits created by actual recycling efforts. Bourtsalas et al.^11^ measured a decrease in global warming potential in the United States from 20 Mt CO_2_-eq in the scenario where 100% of plastics are exported to −11.1 Mt CO_2_-eq in the scenario where 100% of plastics are treated domestically during 2002–2020. Yet, the latter domestic recycling scenario hinges on a hypothetical 50% domestic recycling rate, diverging from the reported recycling rates of the United States.^12,13^ We give an overview of such previous research in Table S1.

Shortcomings are evident in current models assessing the environmental impacts of global plastic waste trade as indicated in the following:

(1) Average domestic treatment assumptions. The treatment impact of imported plastic waste is closely tied to the recycling rate. Usually, due to data constraints, a country’s average treatment mix (recycling, incineration, landfill, etc.) is used as a proxy for the treatment of imported plastic waste.^1,14^ However, this simplification raises two critical concerns. First, recyclability varies between domestic and imported plastic waste. Domestically generated plastic waste is typically collected and sorted from diverse sources, often containing more mixtures and impurities. In contrast, imported plastic waste is usually more concentrated and intended for recycling. Second, trade data from the UN Comtrade indicate that importing countries pay for plastic waste,^15^ suggesting an economic incentive and therefore a reasonable recycling rate to break even. Thus, the actual recycling rate for importers is influenced by both import costs and expected recycling returns, resulting in a rate that typically surpasses the domestic average.^16,17^

(2) Absence of “nontrade” scenarios. Solely quantifying the environmental consequences of treating the traded plastic waste, managed by waste-importing countries, may overlook the environmental impact related to reduced domestic plastic waste treatment in waste-exporting countries.^18^ This impact could be assessed in “nontrade” scenarios with explicit assumptions about how previously exported plastic waste would be managed domestically. Therefore, a thorough evaluation of environmental consequences associated with plastic waste trade should either compare the environmental impacts between trade and “nontrade” scenarios or quantify its net environmental impact.^19^

(3) Carbon-centric metrics: Much of the current literature predominantly focuses on greenhouse gas emissions. However, the end-of-life plastic treatment contributes a mere 10% to the entire life cycle emissions of plastics.^20^ Since plastics contribute to other impacts beyond global warming, a broader spectrum of impact categories deserves exploration and comparison when discussing the plastic waste trade.

In this study, we aim to address these research gaps by quantifying the environmental impact of plastic waste traded among 18 countries in 2022, representing 60% of global plastic waste trade. We use the “required recycling rate” (RRR) to simulate the recycling fate of imported plastic waste, which considers importers’ costs and recycling revenues across countries and plastic waste types. We compare the environmental impacts of trade in 2022 with three “nontrade” counterfactual scenarios, considering varied treatment structures. Using life cycle assessment, we analyze environmental impacts across midpoint and end point categories by treatments and countries. Finally, we discuss the pivotal role of the recycling rate in evaluating the environmental impacts of plastic waste trade and propose refinements for developing the global plastics treaty.

## 2 Methods

### 2.1 Country Coverage

In this work, we selected 18 countries that consistently ranked within the top 80% of either global plastic waste importers or exporters between 2018 and 2022, relying on data from the UN Comtrade database. Trade flows among these countries alone represent 60% of global trade in plastic waste in 2022. The considered countries are further divided into three geographical regions, with Malaysia, Indonesia, Vietnam, Taiwan (China), Japan, and Turkey in Asia; the UK, The Netherlands, Germany, Austria, Belgium, Spain, France, Italy, and Poland in Europe; and the US, Canada, and Mexico in North America (see Table S2).

### 2.2 Plastic Waste Trade Flows

We use UN Comtrade as the data source for four plastic waste types being traded globally in 2022. These include waste plastics of ethylene polymers (waste PE; HS code 391510), of styrene polymers (waste PS; HS code 391520), of vinyl chloride polymers (waste PVC; HS code 391530), and of other plastics (other waste plastics; HS code 391590).^21^ To better understand the varying environmental impacts associated with treating different types of plastic waste, we expand upon the existing traded plastic waste categories. Specifically, we subdivided the plastic waste PE into waste HDPE and waste LDPE, while separating the plastic waste “Others” into waste PET and waste PP. The ratio for splitting is determined by the plastic recycling structure in waste-importing countries, as outlined in Table S3. Comtrade specifies further the “transport mode” used for imports and exports per type of waste plastic. Partially, Comtrade contains imbalances—as for each country pair the imports from, e.g., country B reported by country A may differ from the related exports to A reported by country B. In such instances, we reconcile the average weight value through the following approach: if both trading countries report a transaction, we apply the average value from both countries. Additionally, when an imbalance across transportation modes is reported between two trading countries (e.g., country A reports transactions a1 via land and a2 via sea, while country B reports b1 only via sea), the average value is applied to transactions via the same mode of transport (specifically, the average of a2 and b1). After reconciling the trade reported by importers at 4.10 Mt and by exporters at 4.34 Mt in 2022, the final plastic waste trade amounted to 4.20 Mt.

### 2.3 Required Recycling Rate and Domestic Recycling Rate

As indicated, most previous studies assumed imported plastic waste to be treated similarly to the average treatment mix of domestic plastic waste in the waste-importing country. This assumption is inconsistent, especially for some Asian importers, as the imported plastic waste is often more presorted, resulting in a relatively higher level of concentration compared to their domestically generated plastic waste.^22,23^ In addition, it is paid for by waste-importing countries. To have a continuous incentive for importing plastic waste, there must be a steady and reliable realization of profits by importers. This implies that the returns from selling recycled plastics must at least outweigh the required key costs, which are import prices and recycling costs (including labor costs, electricity costs, and rental payments), along with physical losses throughout the recycling process. Therefore, we model the minimum required recycling rate of imported plastic waste (referred to as RRR hereafter) with a cost-benefit equation (eqs 1–3). The RRR for four original types of plastic waste (refer to PE, PS, PVC, and “Others”) across 18 countries in 2022 (see results in Table S4).
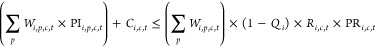
1
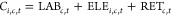
2
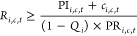
3where *W*_*i*,*p*,*c*,*t*_ indicates the net weight of the imported plastic waste of type *i* (referring to one of four waste plastics documented in the harmonized system (HS): PE, PS, PVC, and others) being exported from country *p* to country *c* in the year *t*; PI_*i*,*p*,*c*,*t*_ indicates the per-unit price of imported plastic waste of type *i* from country *p* to country *c* in the year *t*; *C*_*i*,*c*,*t*_ denotes the operational costs during the mechanical recycling of plastic waste *i* in the importing country *c* for the year *t*, including costs for labor (LAB_*c*,*t*_), electricity (ELE_*i*,*c*,*t*_), and rent (RET_*c*,*t*_) in eq 2. *Q*_*i*_ indicates the physical loss of plastic waste of type *i* during mechanical recycling. *R*_*i*,*c*,*t*_ indicates the recycling rate of imported plastic waste of type *i* in the country *c* of the year *t*; PR_*i*,*c*,*t*_ indicates the per-unit price of recycled plastic of type *i* in the importing country *c* for the year *t*. *c*_*i*,*c*,*t*_ denotes the per-unit operational cost, resulting from dividing *C*_*i*,*c*,*t*_ by ∑_*p*_*W*_*i*,*p*,*c*,*t*_. The calculations of PI_*i*,*p*,*c*,*t*_ and PR_*i*,*c*,*t*_ are explained in detail in the Supporting Information.

The domestic recycling rate for plastic waste primarily focuses on domestically generated waste, occasionally including imported plastic waste depending on the country’s statistics.^24^ The domestic recycling rate is compiled from various sources, along with shares of other treatments for each research country. Information on the shares of recycling, incineration, and landfill for nine European countries is obtained from Plastics Europe (the Association of Plastics Manufacturers in Europe).^25^ Treatment mixes for the USA, Canada, Malaysia, Taiwan (China), and Japan are accessed from governmental or department reports. Additionally, data for Indonesia and Vietnam are derived from research reports conducted by nonprofit organizations. The “average treatment mix” including shares of all treatments of plastic waste for each country is detailed in Table S2 with corresponding references. When multiple data sources had been identified, we computed and applied the average.

### 2.4 Scenario Setting

We conducted four scenarios with different treatment structures to account for variations in handling traded plastic waste, both domestically and abroad (assumed) in 2022 (see Table 1). These scenarios include one trade scenario (TD), reflecting actual trade flows in 2022, and three nontrade scenarios (NT1–NT3), which assume that exported plastic waste is treated domestically with varying recycling rates.

**Table 1:** Scenarios of Plastic Waste Trade in 2022^a^

scenarios	TD	NT1	NT2	NT3
simulated situations	exported waste was transported and treated in waste-importing countries with RRR in 2022	assuming the exported waste was treated domestically in waste-exporting-countries with a 100% recycling rate in 2022	assuming the exported waste was treated domestically in waste-exporting countries with RRR in 2022	assuming the exported waste was treated domestically in waste-exporting countries with the average treatment mix
waste-treating countries	importing countries	exporting countries	exporting countries	exporting countries
share of recycling	RRR across countries and plastic waste types	100%	RRR across countries and plastic waste types	domestic recycling rate
share of other treatments	takes the rest share as same proportion as in the average treatment mix	0	takes the rest share as same proportion as in the average treatment mix	same as in the average treatment mix
international transport included or not	yes	no	no	no

a The “average treatment mix” indicates the shares of domestic plastic waste treatments, including shares for recycling, incineration (with or without energy for recovery), sanitary landfill, unsanitary landfill, open dumping, and open burning, which is detailed in Table S2. The “transport mode” of each transaction is reported in the UN Comtrade database.

### 2.5 Life Cycle Assessment

#### 2.5.1 Goal and Scope

In this study, the goal of conducting an attributional life cycle assessment (LCA) is to evaluate the environmental impacts of plastic waste trade in 2022. The scope of this assessment includes international transport and treatment of exported plastic waste, including seven end-of-life treatments: mechanical recycling, incineration (with and without energy recovery), sanitary landfill, unsanitary landfill, open dumping, and open burning. The boundary for the mechanical recycling process starts from the sorted plastic waste stream to plastics in their primary forms, including pellets, granules, flakes, and similar bulk forms,^26^ as illustrated in Figures S1–S3. Our functional unit for plastic waste treatment involves processing 1 kg of plastic waste, distinguished by six plastic waste types, seven waste treatment methods, and across 18 research countries. Additionally, the functional unit for international transport refers to the transportation of 1 kg of plastic waste for 1 km between trading countries via one of four transport modes: sea, road, air, and railway.

#### 2.5.2 Inventory Analysis

The life cycle inventory (LCI) data were primarily sourced from the commercial Ecoinvent 3.8 cutoff database and the open-access LCA Commons database developed by the United States Department of Agriculture (see Table S5).

The LCI data for mechanical recycling of six plastic waste types was compiled through literature reviews. Inventories were established to cover both lower and upper ranges of resource consumption and residual output. To assess the impact of avoiding virgin plastic production through recycling, we introduce a substitution factor which we multiply with the per-unit impact of virgin plastic production (see Table S5). Each virgin plastic production is linked to two LCIs from the Ecoinvent 3.8 and LCA Commons databases, considering varying geographical coverage in Europe and the USA. We adjusted the original LCI data to incorporate country-specific electricity consumption and electricity production mixes across 18 research countries using electricity market activities from the Ecoinvent 3.8 database (see Table S5). Substitution factors for the six types of recycled plastics primarily consider their mechanical and nonmechanical properties in comparison to their virgin counterparts (see Table S15).

The original LCI data for the incineration of the six plastic waste types is sourced from the Ecoinvent 3.8 database. We further differentiate recovered energy (i.e., electricity and heat generation) from incineration across 18 research countries with references in Table S13. We calculate the avoided net energy generation across countries and plastic waste types by taking into account the efficiency of energy recovery (net energy generation from incinerator) and the ratio of the lower heating values of the specific plastic waste to the general waste (feedstock to incinerator). We used the following equation (eq 4) to quantify the avoided net energy generation among 18 research countries:
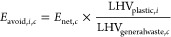
4where *E*_avoid,*c*_ represents the avoided net energy generation (electricity or heat) for incinerating per-unit plastic *i* in country *c*. *E*_net,*c*_ indicates the net energy generation (electricity or heat) for incinerating per-unit general waste in country *c*. LHV_plastic,*i*_ denotes the lower heating value per unit mass of plastic *i*. LHV_generalwaste,*c*_ denotes the lower heating value per unit mass of the general waste in country *c*. *E*_net,*c*_, LHV_plastic,*i*_, and LHV_generalwaste,*c*_ are detailed in Tables S12–S14.

The LCI data for landfill practices for each plastic waste, including open dumping, unsanitary landfill, and sanitary landfill, has been sourced from the Ecoinvent 3.8 database with country-specific electricity. These landfill practices vary in terms of protective measures, encompassing options with or without basic cover, leachate protection, and landfill gas disposal systems.^27^ Additionally, the LCI data for the open burning of each plastic waste is obtained from the Ecoinvent 3.8 database. We refer to Table S5 for details on the LCIs for landfill and open burning.

Four transport modes are recorded in the UN Comtrade bilateral trade database. The transport distance between trading countries via sea, air, and road (including railway) was derived from the CERDI-sea distance database,^28^ the great-circle distance calculation given capital latitude and longitude,^29^ and Google distance matrix API,^30^ respectively. The LCI data for four types of transport are derived from the Ecoinvent 3.8 database, which is shown in Table S5.

#### 2.5.3 Impact Assessment

We evaluated all 18 midpoint and three end point impact categories using the life cycle impact assessment method of ReCiPe (H) V1.13.^31^ In the main text, we present the results for two midpoint impact categories, climate change and marine ecotoxicity, as well as all three end point impact categories, damages to ecosystem quality, human health, and resource availability. The results for the remaining 16 midpoint indicators are presented in Figure S4.

#### 2.5.4 Interpretation

The related calculations were executed using Activity Browser,^32^ open-source software for life cycle assessment (LCA) built on Brightway 2.^33^ The Python script and the related data are publicly accessible on Zenodo at https://zenodo.org/records/10987746.

### 2.6 Sensitivity Analysis

A one-at-a-time sensitivity analysis was conducted to determine how the alteration of seven key parameters affects the environmental impacts across impact categories and scenarios. When changing one parameter at a time, the fluctuation of environmental impacts (lower and upper boundaries) is determined by two parameter values associated with optimistic and pessimistic cases,^34^ which is defined in Table 2.

**Table 2:** Uncertain Parameters in the Sensitivity Analysis

sources of uncertainty	symbols in the sensitivity analysis	uncertain parameters	optimistic	pessimistic
waste treatment structure	P1	required recycling rate (RRR)	highest RRR during 2013–2022 by country and plastic waste type	lowest RRR during 2013–2022 by country and plastic waste type
LCI of waste treatment	P2	substitution factor of recycled plastics	highest substitution factor by plastic waste type through literature review (detailed in Table S15)	lowest substitution factor by plastic waste type through literature review (detailed in Table S15)
LCI of waste treatment	P3	LCI of mechanical recycling (including avoided virgin plastic production)	consuming fewer resources and handling fewer residuals to recycle per unit waste plastics (detailed LCI in Tables S6–S11)	consuming more resources and handling more residuals to recycle per unit waste plastics (detailed LCI in Tables S6–S11)
LCI of waste treatment	P4	LCI of incineration (energy for recovery; including avoided energy production)	consuming less resources and producing more energy for recovery for incinerating per unit waste plastics (detailed LCI in Tables S12–S14)	consuming more resources and producing less energy for recovery for incinerating per unit waste plastics (detailed LCI in Tables S12–S14)
waste treatment structure	P5	share between sanitary landfill, unsanitary landfill, and open dumping in waste treatment	choosing the lowest impact among allocating all the share for sanitary landfill, unsanitary landfill, or open dumping	choosing the highest impact among allocating all the share for sanitary landfill, unsanitary landfill, or open dumping
trade data	P6	share of HDPE and LDPE in the “waste PE” category (HS391510)	choosing the lower impact between assuming all HDPE or all LDPE in this category	choosing the higher impact between assuming all HDPE or all LDPE in this category
trade data	P7	share of PET and PP in the “waste others” category (HS391590)	choosing the lower impact from assuming all PET or all PP in this category	choosing the higher impact from assuming all PET or all PP in this category

## 3 Results

### 3.1 Plastic Waste Trade Flows in 2022

**Figure 1:**
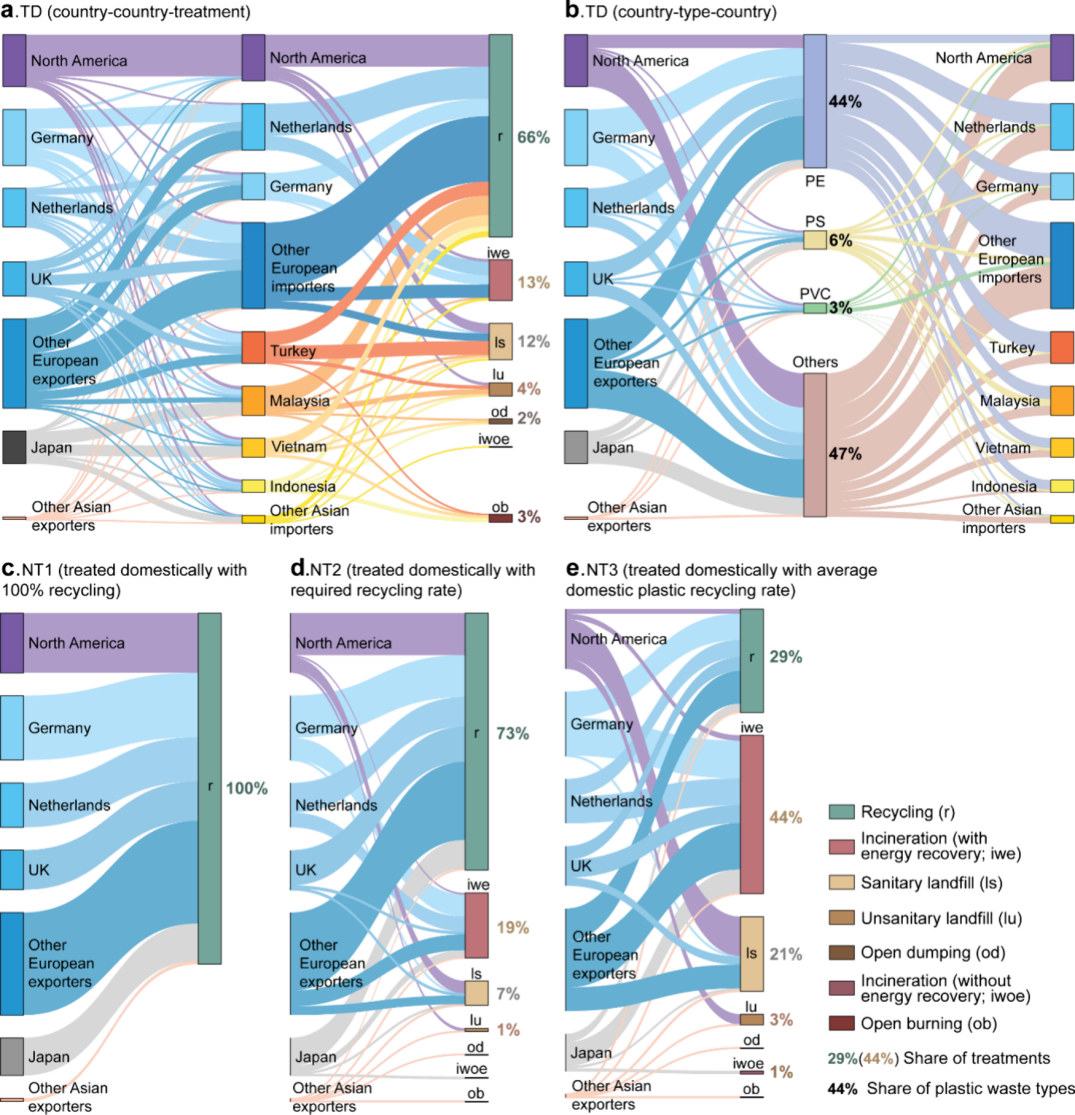
Bilateral plastic waste trade flows in 2022. (a) Distribution by trading countries and end-of-life treatments in the trade scenario. (b) Distribution by plastic waste types in the trade scenario. (c–e) Distribution by end-of-life treatments in three nontrade scenarios. The top importers and exporters are listed individually, with other research countries grouped. “Other European exporters” include Belgium, France, Italy, Spain, Poland, and Austria. “Other Asian exporters” include Turkey, Taiwan (China), Malaysia, Indonesia, and Vietnam. “Other European importers” include the UK, Belgium, France, Italy, Spain, Poland, and Austria. “Other Asian importers” include Japan and Taiwan (China). “North America” includes the USA, Canada, and Mexico.

In the trade scenario (TD), approximately one-third of traded plastic waste ended up in Asian countries in 2022. However, in the three nontrade scenarios that assumed domestic treatment of exported waste, this volume plummets to zero. In total, the trade scenario covers 4.2 Mt of plastic waste exchanged among the selected 18 researched countries (Figure 1a), making up 60% of the total plastic waste trade across 186 countries in 2022. For imports, countries in Europe (9), Asia (6), and North America (3) contributed to a ratio of 3:2:1. However, except for Japan, few Asian countries acted as exporters. The share in exports for other Asian countries accounts for 0.04 Mt or less than 1% in the three nontrade scenarios (Figure 1c–e). A breakdown by plastic type reveals that the majority of traded waste was categorized into groups of “Others” and “PE,” with groups of “PS” and “PVC” making up less than 10% in 2022 (Figure 1b).

In the nontrade scenario NT3, where we assume the exported plastic waste is treated domestically with the average treatment mix, the recycling rate is lowest at 29%, with incineration peaking at 44%. The trade scenario (TD), featuring the RRR to balance importer costs with recycling benefits, yields a recycling rate of 66%. Notably, even with this elevated recycling share, around 3% of the traded waste in TD ends up being open burned due to remaining waste mismanagement shares in certain Asian importing countries. In the nontrade scenario NT2, assuming the exported plastic waste is treated domestically with the RRR, the recycling share increases to 73%, given that RRRs in many European and North American countries are higher than those in the waste-importing countries.

### 3.2 Environmental Impacts of the Plastic Waste Trade

**Figure 2:**
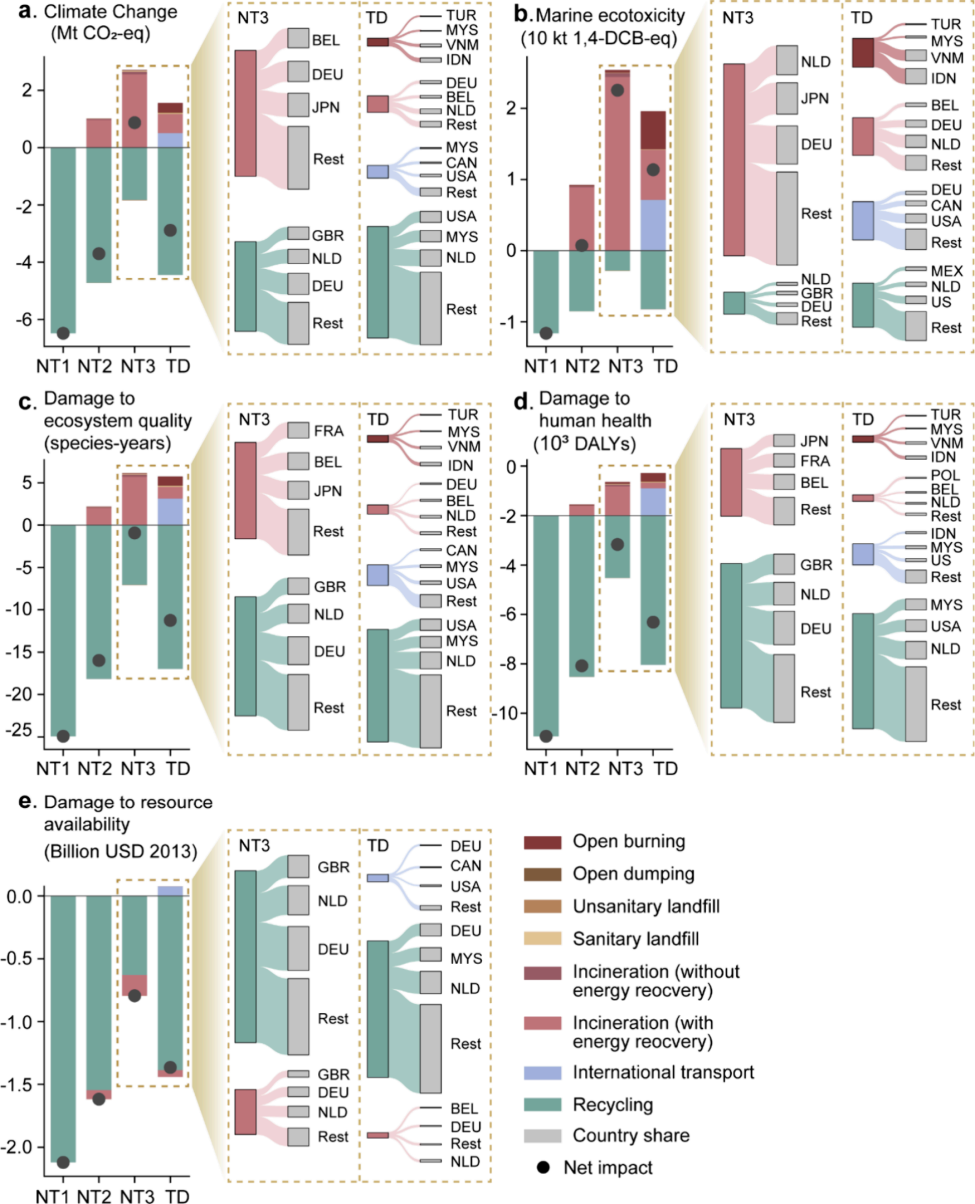
Environmental impacts of plastic waste trade in 2022 under four scenarios by waste treatments (breakdown by the top 3 countries and the rest between NT3 and TD scenarios). The environmental impacts are covered by two midpoint and three end point impact categories: climate change (a), marine ecotoxicity (b), damage to ecosystem quality (c), damage to human health (d), and damage to resource availability (e). Full country names matching their ISO country codes are given in Table S2.

Considering the environmental impacts of international transport and plastic waste treatments, we observed that the trade scenario (TD) generally resulted in lower environmental impacts (or more environmental benefits) compared to treating plastic waste domestically using the average treatment mix (NT3), as depicted in Figure 2. Although overall environmental benefits were evident across various midpoint impact categories among four scenarios, the NT3 scenario stood out for its significant environmental impacts, particularly in climate change and marine ecotoxicity. In Figure 2, we illustrate the impacts of plastic waste trade on climate change, marine ecotoxicity, and three end point impact categories in 2022 across four scenarios. The results of the other 16 midpoint impact categories are presented in Figure S4. Specifically, we highlight the impact difference between the nontrade scenario NT3 and the trade scenario TD at the country level.

Recycling plays a pivotal role in creating environmental benefits in the plastic waste trade. In the context of climate change (Figure 2a), the trade scenario (TD) yielded significant net carbon benefits, primarily derived from plastic waste recycling, amounting to 2.85 Mt in 2022. This equates to roughly 30% of the annual primary PET production across the 30 European Economic Area (EEA) countries.^25^ The impacts of nontrade scenarios hinge on assumed recycling rates. NT1, assuming 100% domestic recycling of exported plastic waste, resulted in an avoided climate change impact of 6.5 Mt CO_2_-eq, doubling the carbon benefits compared to the TD scenario. Conversely, NT3, assuming domestic recycling based on exporting countries’ average treatment mix, resulted in a lower recycling rate (29%) compared to the RRR used in the TD scenario (66%; Figure 1). Consequently, NT3 exhibited the highest climate change impact among scenarios, at 0.94 Mt CO_2_-eq NT2; recycling exported plastic waste domestically using the RRR produced more carbon benefits than the TD scenario, at 3.71 Mt CO_2_-eq, given higher RRR in waste-exporting countries relative to waste-importing ones in 2022. Recycling similarly brought environmental benefits across three end point impact categories in all scenarios (Figure 2c–e). For instance, primarily influenced by the avoided impacts from recycling, the trade scenario reduced damages to ecosystem quality, human health, and resource availability by 12 species-years, 6200 DALYs, and 1.4 billion USD (2013), respectively.

Moreover, a comparison between trade and nontrade scenarios underscores a significant reduction in the environmental impact stemming from incineration. Typically, incineration (with energy recovery) rates are higher in most European countries compared to the global average.^35^ This explains why treating exported plastic waste domestically with the average treatment mix (NT3) in 2022 would lead to a 31% increase in total incineration than in the trade scenario, as highlighted in Figure 1 (a and e), given that most exporters are European countries. Consequently, incineration (with energy recovery) accounts for nearly all environmental burdens in the NT2 and NT3 scenarios (Figure 2a–e). However, as increased plastic waste was sent for recycling in waste-importing countries in the trade scenario (TD) in 2022, the environmental impact from incineration was reduced across all impact categories. For instance, the impact of incineration on climate change and marine ecotoxicity decreased by nearly 70% when comparing NT3 and TD scenarios (Figure 2a,b).

Despite the reduced impact of incineration in the trade scenario, there was still an environmental risk from mismanaged treatments in waste-importing countries. In the TD scenario, only 3% of total traded plastic waste underwent open burning in countries like Indonesia, Vietnam, Malaysia, and Turkey. However, this small fraction contributed disproportionately to climate change and marine ecotoxicity impacts, accounting for 6% and 26% in 2022, respectively (Figure 2a,b). Specifically, approximately 0.13 Mt or 3% of all traded plastic waste underwent open burning in 2022 in the TD scenario, distributed among those countries. Considering different contributions to climate change across treatments plus international transport, the impact of open burning on climate change effectively doubled relative to its physical trade flow proportion in 2022, equivalent to 0.38 Mt CO_2_-eq. The same amplified environmental impact of open burning was observed in marine ecotoxicity.

**Figure 3:**
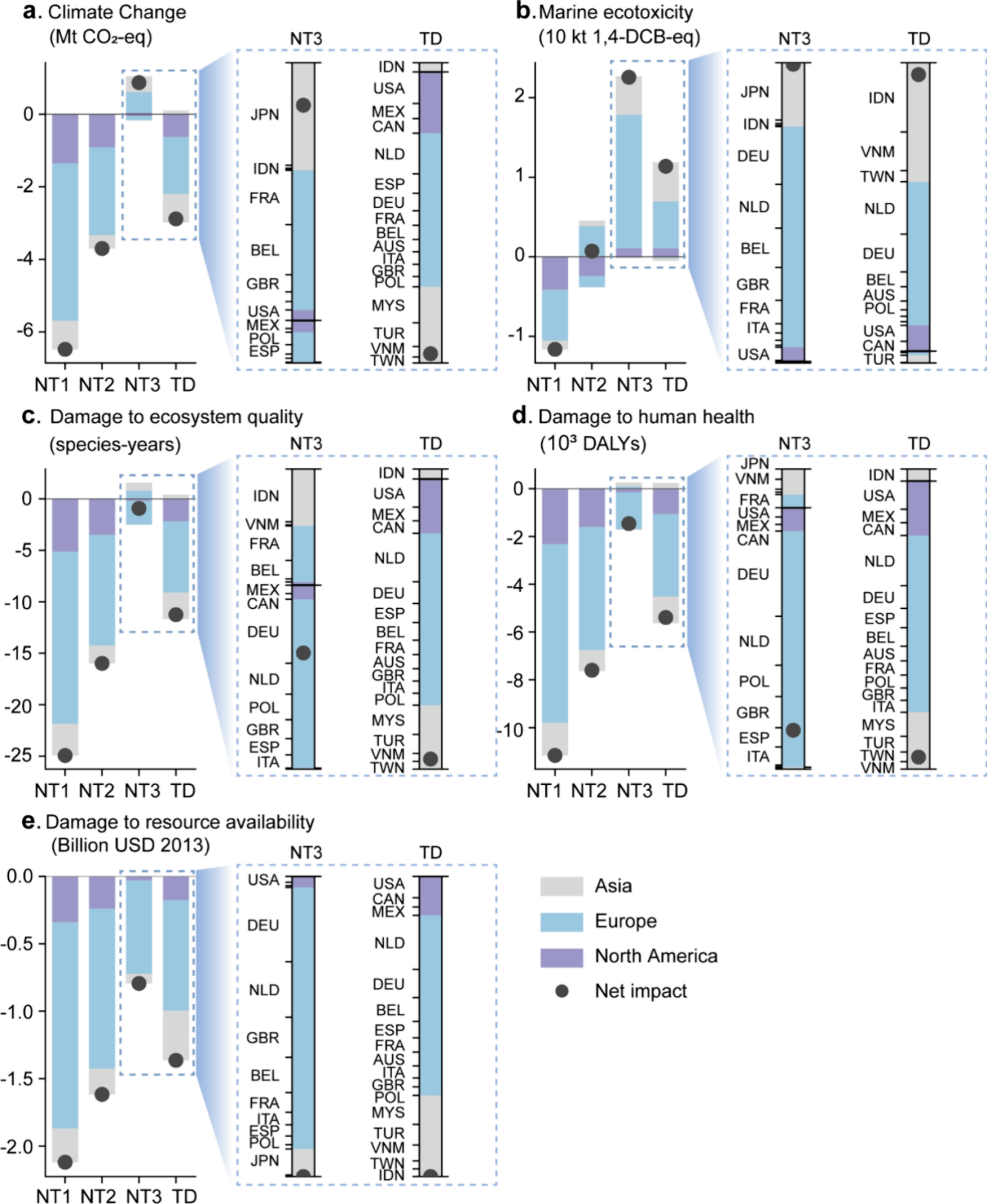
Environmental impacts of the plastic waste trade in 2022 under four scenarios by regions and countries (breakdown by country in NT3 and TD scenarios). The environmental impacts are covered by two midpoint and three end point impact categories: climate change (a), marine ecotoxicity (b), damage to ecosystem quality (c), damage to human health (d), and damage to resource availability (e). To avoid overlapped labels, countries with relatively small proportions are removed from the figure. Full country names matching their ISO country codes are given in Table S2. Since the original LCA results are aggregated as either positive (stacked above zero) or negative (stacked below zero) values at both treatment and region levels (see Figure 2 and here), the length of the bar representing each scenario varies. However, the net environmental impact remains consistent, as indicated by the position of the black dot, regardless of bar length.

In Figure 3, we analyze the diverse environmental consequences of plastic waste trade across regions, examining impacts at both regional and country levels. It is important to note that we adopted a “producer’ view to allocate environmental impacts and benefits, both regionally and nationally. This allocation attributes environmental responsibility to countries that initially import plastic waste for recycling into primary plastics, while excluding other countries that may subsequently import and use these recycled primary plastics. Notably, compared to Asian importers, European importers accounted for the most significant environmental benefits in the trade scenario in 2022. Regarding climate change, the plastic waste trade scenario characterized by RRR yielded carbon benefits of 0.8 Mt CO_2_-eq for four Asian countries (Malaysia, Turkey, Vietnam, and Taiwan (China)), whereas no carbon benefits were observed in those countries in the NT3 scenario. Similarly, compared to the NT3 scenario, Asian importers gained the avoided damages to ecosystem quality, human health, and resource availability at 3.4 species-years, 1270 DALYs, and 0.3 billion USD (2013), respectively, in the trade scenario in 2022. However, European countries still accounted for the most environmental benefits in the trade scenario, with avoided damages to ecosystem quality, human health, and resource availability at 7 species-years, 3500 DALYs, and 0.8 billion USD (2013).

### 3.3 Sensitivity Analysis

**Figure 4:**
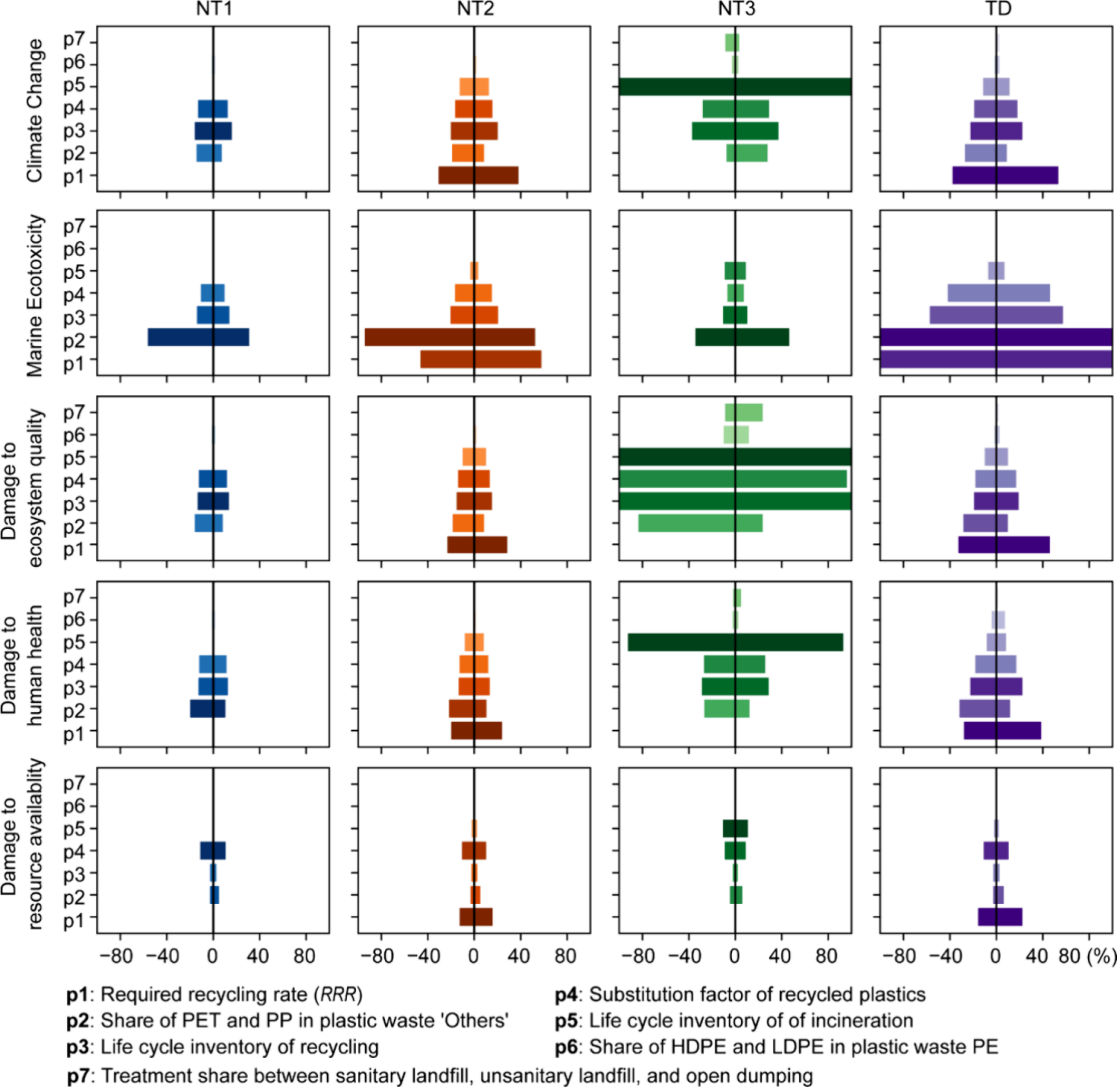
Sensitivity analysis for the selected impact categories under four scenarios. The variance exceeds or equals 100% as the bar reaches its end point. Additional results for the remaining 16 midpoint impact categories are provided in Figures S5 and S6. The length of the horizontal bars reflects the range of sensitivity results obtained from pessimistic and optimistic cases. The color depth indicates the relative sensitivity levels among the seven parameters.

We identified seven key parameters that could impact the environmental outcomes across impact categories and scenarios, focusing on waste treatment structure, life cycle inventory (LCI) of waste treatment, and trade data (detailed in Table 2). These parameters and their sensitivity analysis results are depicted in Figure 4. For each parameter, two limitation values representing pessimistic and optimistic environmental impacts were chosen, influencing the length of each parameter bar as shown in Figure 4.

The most fluctuations of environmental impacts remain in NT3 and TD scenarios. In the trade scenario (TD), fluctuations in environmental impacts roughly span from −40% to 40%, with the recycling recovery rate (RRR) and LCI of recycling emerging as the most influential parameters. The largest fluctuation under the TD scenario is observed in marine ecotoxicity, where fluctuations resulting from RRR and the uncertain share of PET and PP in plastic waste “Others” reach the limitation bound of 100%. Meanwhile, the NT3 scenario, characterized by a high proportion of plastic waste incineration, demonstrates heightened sensitivity to the LCI of incineration compared to other parameters. Conversely, fluctuations in environmental impacts are relatively narrowed in scenarios NT1 and NT2, spanning within ±20%. In the NT2 scenario, RRR emerges as the most sensitive parameter, aligning with the TD scenario where RRR plays a significant role. In contrast, in the NT1 scenario, assumed 100% domestic recycling leads to fluctuations in environmental impacts primarily influenced by recycling-related parameters, including the share of PET and PP in plastic waste “Others” and LCI of recycling.

## 4 Discussion

The net environmental impacts of the plastic waste trade heavily rely on underlying recycling rate assumptions for waste-treating countries. In our study, when factoring in RRR for importing countries, we find that the trade scenario in 2022 contributed to emissions reductions of 2.85 Mt CO_2_-eq. This sharply contrasts with Wen et al.’s estimated increase of 0.13 Mt CO_2_-eq in climate change,^1^ derived from their use of domestic average recycling rates for imported plastics in their “2018 trade scenario,” which also featured a 25% lower plastic trade volume compared to our work. The key discrepancy lies in the assumed recycling rates: applying average domestic rates to imported waste plastics overlooks the fact that importers pay for and invest in recycling. Importers bear the costs of imported plastic waste and recycling, and only when achieving a recycling rate (the RRR) that generates revenues equal to these costs do such imports become economically viable.^16^ Typically, the RRR surpasses average domestic recycling rates,^17^ resulting in increased recycled plastics output, reduced incineration, and, ultimately, decreased environmental impacts. This dynamic is explored in our study through a comparison of NT3 and TD scenarios.

Instead of advocating for policies that simply prevent plastic waste from being sent to global south countries, we propose a more nuanced approach: directing plastic waste away from importers with lower recycling rates for imported plastic. While a global south country may indeed have a lower recycling rate for domestic plastic waste, this does not necessarily apply to its imported plastic waste, which can be recycled up to 66% on average in the trade scenario TD (Figure 1a). Our findings also indicate the preference for domestic treatment without the trade if specific recycling rates can be attained. The NT2 scenario, for instance, assuming exported plastic waste undergoes treatment domestically using RRR, yields greater environmental benefits than if treated in waste-importing countries (TD) in 2022, with 73% of traded plastic waste undergoing recycling. Similarly, the ideal NT1 scenario illustrates that achieving 100% recycling domestically maximizes environmental benefits. Therefore, we advocate directing plastic waste to locations where the highest rates of recycled plastics can be achieved.

In ongoing UN negotiations, 175 nations aim to create a binding agreement to tackle plastic pollution comprehensively by 2024.^36^ This encompasses the entire plastic life cycle, including design, production, and disposal. Rather than solely considering waste plastics treatment as end-of-life measures,^37^ we emphasize that it is crucial to recognize their value as a feedstock for secondary plastic production. Especially, much of the high-quality waste plastics presorted for recycling are redistributed via international trade,^38^ forming the backbone of global secondary plastic production. Consequently, we suggest that regulating the trade of plastic waste to ensure purity, recyclability, and traceability should be identified as a critical source-control measure within the plastic treaty framework, transcending its traditional classification as a mere end-of-life issue.^39^ Moreover, investing solely in waste treatment infrastructure may not adequately address plastic pollution in countries of the Global South, which primarily serve as waste importers. The economic dynamics of waste imports often resulted in a situation where imported plastic waste occupied the capacity of domestic waste treatment facilities, potentially at the expense of locally generated waste.^40^ Thus, rather than indiscriminately constructing new waste treatment facilities in Global South countries, emphasis should be placed on optimizing their domestic sorting and collection systems to fully capitalize on their domestic plastic waste.

Certain impacts of the plastic waste trade are not covered in this work, including plastic leakage and related microplastic issues, which pose threats to both human health and animal welfare.^41^ While some research has explored the influence of plastic waste trade on plastic leakage into aquatic environments,^42^ our findings indicated that the impact of traded plastic waste on marine leakage and microplastics is likely less significant in comparison with domestically treated waste. According to the UN Comtrade, globally traded plastic waste accounted for 7 million tonnes in 2022 (after balance),^38^ representing 2% of the total plastic waste generated worldwide, which amounts to approximately 350 million tonnes.^43^ One reason for the low observed ratio is that it is mostly recycled waste that is traded, whereas less than 10% of the world’s generated plastic is recycled.^44^ Therefore, compared to imported plastic waste, domestically generated waste has a higher likelihood of being mismanaged and leaked into the environment due to inadequate sorting and recycling systems.^40^ However, it is important to note that recycling processes^45^ and illegal trade,^46^ which are not investigated in this work, can still contribute to environmental leakage, highlighting the need for further examination.

## Data Availability

All gathered data and the Python scripts for analyzing and plotting the results are publicly accessible on Zenodo at https://zenodo.org/records/10987746.

## Supporting Information Available

Expanded methodology description on calculating the required recycling rate across countries and plastic waste types; related data on the domestic average plastic treatment mix across countries, the share of plastic types in recycling across countries, the required recycling rate across countries and plastic waste types, life cycle inventory, recovered electricity and heat from municipal waste incineration across countries, substitution factors of secondary plastic to primary plastic regarding technical properties, etc; supplementary figures on the system boundaries of mechanical recycling in life cycle assessment across plastic waste types, environmental impacts across the rest of the impact categories, and sensitivity analysis of the rest of the impact categories (PDF)
