# Supplementary material for: Traded Plastic, Traded Impacts? Designing Counterfactual Scenarios to Assess Environmental Impacts of Global Plastic Waste Trade
Source: Environ Sci Technol. 2024 May 10;58(20):8631–42. doi: 10.1021/acs.est.4c02149 (PMC11112728; doi:10.1021/acs.est.4c02149)
Supplement: Supplementary file 1 — es4c02149_si_001.pdf [file es4c02149_si_001.pdf]

Supporting information for

Traded plastic, traded impacts? Designing counterfactual scenarios to assess environmental impacts of global plastic waste trade

Kai Li [1]*, Hauke Ward [1], Hai Xiang Lin [1,2], and Arnold Tukker [1,3]

[1] Institute of Environmental Sciences (CML), Leiden University, 2333 CC, Leiden, The Netherlands

[2] Delft Institute of Applied Mathematics, Delft University of Technology, 2628 CD, Delft, The Netherlands

[3] Netherlands Organization for Applied Scientific Research TNO, 2595 DA, The Hague, The Netherlands

* Corresponding author: Kai Li, k.li@cml.leidenuniv.nl

Number of pages: 37

Number of notes: 1

Number of tables: 15

Number of figures: 6

Contents

1. Supplementary note 3

1. Unit prices of plastic waste and recycled plastic across countries and types 3

2. Supplementary table 4

3. Supplementary figure 25

4. References 33

1. Supplementary note

1. Unit prices of plastic waste and recycled plastic across countries and types

Both unit prices for plastic waste and recycled plastic are determined by dividing the total trade values and the total net weights between trading countries using the UN Comtrade database in 2022[1]. As no separate commodity is reported under 'recycled plastics' in the database, we instead use the corresponding primary plastics as an alternative, where the secondary plastics are included. PE is mapped to HDPE and LDPE, and 'Others' is mapped to PET and PP. The split ratio across countries is shown in Table S3. Besides, to ensure the importing costs of transport and insurance are shouldered by importers, the trade value of imported plastic waste adopts a CIF price, while FOB (free on board) pricing is employed for the trade value of exported primary plastic. The unit price of imported plastic waste and primary plastic are calculated as follows:

$$PI_{i,c,t} = \frac{\sum_p V_{i,p,c,t}}{\sum_p W_{i,p,c,t}} \tag{1}$$

$$PR_{i,c,t} = \begin{cases} \frac{\sum_p V_{PS,p,c,t}}{\sum_p W_{PS,p,c,t}} \left(or\ \frac{\sum_p V_{PVC,p,c,t}}{\sum_p W_{PVC,p,c,t}} \right) & \text{if } i \text{ is PS } (or \text{ PVC}) \\ \frac{\sum_p V_{LDPE,p,c,t}}{\sum_p W_{LDPE,p,c,t}} \times r_{LDPE,c} + \frac{\sum_p V_{HDPE,p,c,t}}{\sum_p W_{HDPE,p,c,t}} \times r_{HDPE,c} & \text{if } i \text{ is PE} \\ \frac{\sum_p V_{PET,p,c,t}}{\sum_p W_{PET,p,c,t}} \times r_{PET,c} + \frac{\sum_p V_{PP,p,c,t}}{\sum_p W_{PP,p,c,t}} \times r_{PP,c} & \text{if } i \text{ is Others} \end{cases} \tag{2}$$

where $V_{i,p,c,t}$ and $W_{i,p,c,t}$ indicate the trade value and net weight of imported plastic waste of type i from the country p to country c in the year t; $V_{ps,p,c,t}$ and $W_{ps,p,c,t}$ (also subscripts for PVC, HDPE, LDPE, PET, and PP) indicate the trade value and net weight of six types of primary plastic exported from country c to country p in the year t, respectively. By combining HDPE and LDPE as PE, PET and PP as 'others' with the share factors of $r_{HDPE,c}$, $r_{LDPE,c}$, $r_{PET,c}$, and $r_{PP,c}$ in the country c, six primary plastics are mapped to four plastic waste types.

2. Supplementary table

Table S1. The selected research on quantifying the environmental impacts of plastic waste trade.

Research	Main results	Highlights	limitations
Wen, Xie, Chen and Dinga [2]	China's plastic waste import ban significantly increased four midpoint indicators of environmental impact globally.	• The net environmental impact between waste treated domestically and aboard.	• Unspecified plastic waste (mixture) in LCA<br>• Imported plastic waste treated as a national treatment mix
Bourtsalas, Yepes and Tian [3]	The US could achieve carbon savings of 36 million tons during 2002-2020 if exported plastic waste had been processed domestically.	• Detailed trade flow data at the state level.<br>• Scenario-based recycling rates spanning 0%-8.7%-50%.	• Uniform treatment setting for importing countries outside the USA.
Liu, Liu, Walker, Adams and Zhao [4]	Global plastic waste trade had a net emission reduction of 8.27 Mt CO_2-eq in 2012.	• Linking environmental input-output analysis.	• Without reference to a no-trade scenario.
Ren, Shi, Bardow, Geyer and Suh [5]	China's plastic waste import ban may exacerbate environmental impacts both in China and globally.	• Specified plastic waste (PET) in LCA	• Considered recycling and landfill only.<br>• Uniform treatment setting for importing countries outside China.
Sun and Tabata [6]	A post-ban carbon emission surge in both China and Japan.	• Country-specific life cycle inventory data	• Considered recycling only.

Table S2. The domestic average plastic treatment mix among 18 research countries (unit: %).

Country (ISO3 code)	Region	Year	Recycling	Incineration (with energy recovery)	Incineration (without energy recovery)	Sanitary landfill	Unsanitary landfill	Open dumping	Open burning
USA (USA)	North America	2018	8.6 [7]	15.8 [7]		75.6 [7]			
		2021	5.5 [7]	12.5 [7]		82 [7]			
Canada (CAN)	North America	2018	9 [8]	4 [8]		87 [8]			
		2019	11 [9]	6 [9]		83 [9]			
Mexico (MEX)	North America	2021	9.6 [10]				90.4 [10]		
		2022	6 [11]				94 [11]		
United Kingdom (GBR)	Europe	2018	32.0 [12]	45.9 [12]		22.1 [12]			
		2020	37.0 [13]	44.0 [13]		19.0 [13]			
Netherlands (NLD)	Europe	2018	33.9 [12]	66.1 [12]					
		2020	45.0 [13]	55.0 [13]					
Germany (DEU)	Europe	2018	38.7 [12]	60.3 [12]		1.0 [12]			
		2020	42.0 [13]	57.0 [13]		1.0 [13]			
Austria (AUT)	Europe	2018	29.4 [12]	70.6 [12]					
		2020	31.0 [13]	69.0 [13]					
Belgium (BEL)	Europe	2018	34.0 [12]	63.9 [12]		2.1 [12]			
		2020	39.0 [13]	59.0 [13]		2.0 [13]			
Spain (ESP)	Europe	2018	41.8 [12]	19.2 [12]		39.0 [12]			
		2020	43.0 [13]	21.0 [13]		36.0 [13]			
France (FRA)	Europe	2018	24.0 [12]	42.8 [12]		33.2 [12]			
		2020	25.0 [13]	44.0 [13]		31.0 [13]			
Italy (ITA)	Europe	2018	31.1 [12]	32.8 [12]		36.1 [12]			
		2020	34.0 [13]	34.0 [13]		32.0 [13]			
Poland (POL)	Europe	2018	27.3 [12]	30.2 [12]		42.5 [12]			
		2020	27.0 [13]	32.0 [13]		41.0 [13]			
Malaysia (MYS)	Asia	2019	24.0 [14]	3.0 [14]		10.0 [14]	42.0 [14]	16.0 [14]	5.0 [14]
		2019	21.0 [14]	3.0 [14]		10.0 [14]	44.0 [14]	17.0 [14]	5.0 [14]
Indonesia (IDN)	Asia	2017	11.0 [15]			22.0 [15]	10.0 [15]	5.0 [15]	52.0 [15]
		2019	10.0 [16]			23.0 [16]	10.0 [16]	5.0 [16]	52.0 [16]
Vietnam (VNM)	Asia	2018	15.0 [17]			20.0 [17]	6.0 [17]	27.0 [17]	32.0 [17]
		2022	10.0 [18]			21.0 [18]	7.0 [18]	28.0 [18]	34.0 [18]
Taiwan	Asia	2018	31.0 [19]	67.6[19]		1.4[19]			

(China, TWN)		2019	35.1 [19]	63.6 [19]		1.4 [19]			
		2020	34.0 [19]	64.2 [19]		1.8 [19]			
		2021	26.5 [19]	68.7 [19]		4.8 [19]			
Japan (JPN)	Asia	2018	23.0 [20]	61.0 [20]	8.0 [20]	8.0 [20]			
		2019	22.0 [21]	64.0 [21]	8.0 [21]	6.0 [21]			
		2020	21.0 [22]	65.0 [22]	8.0 [22]	6.0 [22]			
		2021	21.0 [23]	66.0 [23]	8.0 [23]	5.0 [23]			
Turkey (TUR)	Asia	2021	22.0 [24]			61.0 [25]	16.0 [25]		1.0 [25]
		2023	30.0 [26]			55.0 [25]	14.0 [25]		1.0 [25]

Table S3. The share of plastic types in recycling across countries. Research countries without data instead use regional averages.

Country	Share of PET recycling	Share of PP recycling	Share of HDPE recycling	Share of LDPE recycling
Malaysia[27]	29 (2019)	5 (2019)	52 (2019)	8 (2019)
Vietnam (Hanoi) [28]	42 (2022)	-	30 (2022)	1 (2022)
Indonesia (Greater Jakarta) [28]	65 (2020)	11 (2020)	11 (2020)	5 (2020)
Indonesia (Makassar) [28]	70 (2020)	10 (2020)	10 (2020)	5 (2020)
Taiwan, China[29]	55 (2019)	21 (2019)	-	-
Japan[23]	30 (2021)	23 (2021)	20 (PE in total; 2021)	20 (PE in total; 2021)
United Kingdom[30]	34 (2017)	7 (2017)	26 (2017)	3 (2017)
USA[31]	31 (2017)	2 (2017)	20 (2017)	11 (2017)
Spain[32]	22 (2011)	4 (2011)	24 (2011)	29 (2011)
Germany[33]	13 (2017)	26 (2017)	22 (2017)	20 (2017)
EU27[34]	22 (2019)	16 (2019)	18 (2019)	21 (2019)

Table S4. The required recycling rates across 18 research countries and four plastic waste types.

Country	Others	PE	PS	PVC
Austria	82.07%	73.67%	59.95%	80.79%
Belgium	87.12%	67.47%	53.06%	93.64%
Canada	66.40%	66.42%	88.27%	88.48%
France	88.95%	87.22%	41.73%	79.28%
Germany	65.22%	64.80%	63.74%	81.69%
Indonesia	71.99%	39.03%	33.33%	64.20%
Italy	82.60%	74.84%	70.83%	63.28%
Japan	82.07%	73.67%	59.95%	80.79%
Malaysia	70.60%	63.20%	67.24%	62.86%
Mexico	76.23%	43.46%	49.92%	65.74%
Netherlands	77.99%	59.34%	62.61%	77.37%
Taiwan (China)	71.64%	55.94%	55.97%	56.40%
Poland	56.98%	48.36%	34.88%	67.75%
Spain	84.27%	70.02%	56.02%	84.02%
Turkey	46.88%	42.25%	51.22%	47.70%
USA	65.73%	80.48%	58.90%	81.07%
United Kingdom	88.36%	92.01%	71.65%	86.24%
Vietnam	70.70%	49.24%	61.71%	67.12%

Table S5. Original unit processes and adjustments in LCI databases of Ecoinvent 3.8 and LCA Commons. 'Row' is short for 'Rest of the world'.

Stages	Unit process name	LCI database	Coverage	Adjustment
Transport	Transport, freight, sea, container ship	Ecoinvent 3.8	Global	-
Transport	Transport, freight, aircraft, all distances to generic market for transport, freight, aircraft, unspecified	Ecoinvent 3.8	Global	-
Transport	Market for transport, freight, lorry, unspecified	Ecoinvent 3.8	Europe	-
Transport	Market for transport, freight, lorry, unspecified	Ecoinvent 3.8	RoW	-
Transport	Market for transport, freight train	Ecoinvent 3.8	Europe	Combine LCI of diesel and electricity trains with regional share
Transport	Market for transport, freight train	Ecoinvent 3.8	US	
Transport	Market for transport, freight train	Ecoinvent 3.8	RoW	
Avoided HDPE production	Polyethene production, high density, granulate	Ecoinvent 3.8	Europe	Country-specific electricity
Avoided HDPE production	Polyethene, high density, HDPE, virgin resin, at plant	LCA Commons	US	Country-specific electricity
Avoided LDPE production	Polyethene production, low density, granulate	Ecoinvent 3.8	Europe	Country-specific electricity
Avoided LDPE production	Polyethene, low-density, LDPE, virgin resin, at plant	LCA Commons	US	Country-specific electricity
Waste PE sanitary landfill	Treatment of waste polyethene, sanitary landfill	Ecoinvent 3.8	Europe	Country-specific electricity
Waste PE sanitary landfill	Treatment of waste polyethene, sanitary landfill	Ecoinvent 3.8	RoW	Country-specific electricity

Waste PE unsanitary landfill	Treatment of waste polyethene, unsanitary landfill	Ecoinvent 3.8	Region-specific	-
Waste PE open dumping	Treatment of waste polyethene, open dump	Ecoinvent 3.8	Region-specific	-
Waste PE open burning	Treatment of waste polyethene, open burning	Ecoinvent 3.8	Global	-
Avoided PS production	Polystyrene production, expandable	Ecoinvent 3.8	Europe	-
Avoided PS production	Polystyrene, expanded, EPS, virgin resin; batch suspension polymerization; industry average, at plant	LCA Commons	US	-
Waste PS sanitary landfill	Treatment of waste polystyrene, sanitary landfill	Ecoinvent 3.8	Europe	Country-specific electricity
Waste PS unsanitary landfill	Treatment of waste polystyrene, unsanitary landfill	Ecoinvent 3.8	Region-specific	-
Waste PS open dumping	Treatment of waste polystyrene, open dump	Ecoinvent 3.8	Region-specific	-
Waste PS open burning	Treatment of waste polystyrene, open burning	Ecoinvent 3.8	Global	-
Avoided PVC production	Polyvinylchloride production, suspension polymerisation	Ecoinvent 3.8	Europe	Country-specific electricity
Avoided PVC production	Polyvinyl chloride, PVC, virgin resin; at plant	LCA Commons	US	Country-specific electricity
Waste PVC sanitary landfill	Treatment of waste polyvinylchloride, sanitary landfill	Ecoinvent 3.8	Europe	Country-specific electricity

Waste PVC sanitary landfill	Treatment of waste polyvinylchloride, sanitary landfill	Ecoinvent 3.8	RoW	Country-specific electricity
Waste PVC unsanitary landfill	Treatment of waste polyvinylchloride, unsanitary landfill	Ecoinvent 3.8	Region-specific	-
Waste PVC open dumping	Treatment of waste polyvinylchloride, open dump	Ecoinvent 3.8	Region-specific	-
Waste PVC open burning	Treatment of waste polystyrene, open burning	Ecoinvent 3.8	Global	-
Avoided PET production	Polyethene terephthalate production, granulate, amorphous	Ecoinvent 3.8	Europe	Country-specific electricity
Avoided PET production	Polyethene terephthalate, PET, virgin resin, at plant	LCA Commons	US	Country-specific electricity
Waste PET sanitary landfill	Treatment of waste polyethene terephthalate, sanitary landfill	Ecoinvent 3.8	Europe	Country-specific electricity
Waste PET sanitary landfill	Treatment of waste polyethene terephthalate, sanitary landfill	Ecoinvent 3.8	RoW	Country-specific electricity
Waste PET unsanitary landfill	Treatment of waste polyethene terephthalate, unsanitary landfill	Ecoinvent 3.8	Region-specific	-
Waste PET open dumping	Treatment of waste polyethene terephthalate, open dump	Ecoinvent 3.8	Region-specific	-
Waste PET open burning	Treatment of waste polyethene terephthalate, open burning	Ecoinvent 3.8	Global	-
Avoided PP production	Polypropylene production, granulate	Ecoinvent 3.8	Europe	Country-specific electricity

Avoided PP production	Polypropylene, PP, virgin resin, at plant	LCA Commons	US	Country-specific electricity
Waste PP sanitary landfill	Treatment of waste polypropylene, sanitary landfill	Ecoinvent 3.8	Europe	Country-specific electricity
Waste PP sanitary landfill	Treatment of waste polypropylene, sanitary landfill	Ecoinvent 3.8	RoW	Country-specific electricity
Waste PP unsanitary landfill	Treatment of waste polypropylene, unsanitary landfill	Ecoinvent 3.8	Region-specific	-
Waste PP open dumping	Treatment of waste polypropylene, open dump	Ecoinvent 3.8	Region-specific	-
Waste PP open burning	Treatment of waste polypropylene, open burning	Ecoinvent 3.8	Global	-

Table S6. Life cycle inventories for mechanical recycling of high density polyethene (HDPE) with lower and upper ranges. The original life cycle inventory is derived from Civancik-Uslu, Nhu, Van Gorp, Kresovic, Larrain, Billen, Ragaert, De Meester, Dewulf and Huysveld [35]. For other adjustments see references.

	Product	Low	Upper
Input	Sorted waste polyethene, high density	1.00 kg	*
	Electricity, medium voltage	0.30 kWh[36, 37]	0.55 kWh[35, 38, 39]
	Heat (co-generated with power)	0.59 MJ	*
	Tap water	0.28 kg[36]	0.39 kg[40, 41]
Output	Recycled high density polyethene granulate	0.92 kg	0.80 kg
	Municipal solid waste	0.08 kg[42]	0.20 kg[43, 44]

*As the same value as in the low impact.

Table S7. Life cycle inventories for mechanical recycling of low density polyethene (LDPE) with lower and upper ranges. The original life cycle inventory is derived from Civancik-Uslu, Nhu, Van Gorp, Kresovic, Larrain, Billen, Ragaert, De Meester, Dewulf and Huysveld [35]. For other adjustments see references.

	Product	Low	Upper
Input	Sorted waste polyethene, low density	1.00 kg	*
	Electricity, medium voltage	0.30 kWh[36, 40, 43]	0.76kWh[37, 39]
	Heat (co-generated with power)	0.59 MJ	*
	Tap water	0.28 kg[36]	0.34 kg[43]
Output	Recycled low density polyethene granulate	0.92 kg	0.80 kg
	Municipal solid waste	0.08 kg[42]	0.20 kg[43, 44]

* As the same value as in the low impact.

Table S8. Life cycle inventories for mechanical recycling of polystyrene (PS) with lower and upper ranges. The original life cycle inventory is derived from Civancik-Uslu, Nhu, Van Gorp, Kresovic, Larrain, Billen, Ragaert, De Meester, Dewulf and Huysveld [35]. For other adjustments see references.

	Product	Low	Upper
Input	Sorted waste polystyrene	1.00 kg	*
	Electricity, medium voltage	0.29 kWh[36]	0.50 kWh[38, 39]
	Heat (co-generated with power)	0.14MJ	*
	Tap water	0.15 kg	*
Output	Polystyrene, granulate, recycled	0.89 kg	0.87 kg
	Municipal solid waste	0.11 kg	0.13 kg[43]

* As the same value as in the low impact.

Table S9. Life cycle inventories for mechanical recycling of polyvinyl chloride (PVC) with lower and upper ranges. The original life cycle inventory is derived from Ye, Qi, Hong and Ma [45]. For other adjustments see references.

	Product	Low	Upper
Input	Sorted waste polyvinyl chloride	1 kg	*
	Electricity, medium voltage	0.38 kWh	0.47 kWh[38, 46]
	Natural gas	0.045 m^3	*
	Tap water	0.29 kg	*
Output	Polyvinyl chloride, granulate, recycled	0.91 kg	0.89 kg
	Municipal solid waste	0.09 kg	0.11 kg[47]
	Hazardous waste, for incineration	0.01 kg	*
	Sulphur dioxide	4.53×10^{-5} kg	*
	Nitric oxide	1.4×10^{-4} kg	*
	Particulates < 2.5 um	1.61×10^{-4} kg	*
	Xylene	7×10^{-8} kg	*
	NMVOC	2.54×10^{-4} kg	*
	Suspended solids	1.52×10^{-5} kg	*
	BOD5. Biological Oxygen Demand	1.23×10^{-5} kg	*
	Ammonia	3.32×10^{-6} kg	*
	COD, Chemical Oxygen Demand	1.08×10^{-4} kg	*

* As the same value as in the low impact.

Table S10. Life cycle inventories for mechanical recycling of polyethene terephthalate (PET) with lower and upper ranges. The original life cycle inventory is derived from Ecoinvent 3.8 [40]. For other adjustments see references.

	Product	Low	Upper
Input	Sorted waste polyethene terephthalate	1 kg	*
	Electricity, medium voltage	0.29 kWh[36]	0.50 kWh[39]
	Natural gas	5.1×10^{-5} m^3	*
	Diesel	7.5×10^{-4} kg	*
	Tap water	0.28 kg[36]	0.39 kg[40, 41]
	Organic chemical	0.00673 kg	*
	Sodium hydroxide, without water, in 50% solution state	0.0047 kg	*
Output	Polyethene terephthalate, granulate, recycled	0.85 kg	0.76 kg
	Municipal solid waste	0.15 kg[42]	0.24 kg[37]

* As the same value as in the low impact.

Table S11. Life cycle inventories for mechanical recycling of polypropylene (PP) with lower and upper ranges. The original life cycle inventory is derived from Ecoinvent 3.8 [40]. For other adjustments see references.

	Product	Low	Upper
Input	Sorted waste polypropylene	1 kg	*
	Electricity, medium voltage	0.29 kWh[36, 43]	0.55 kWh[35, 39]
	Heat (co-generated with power)	0.097 MJ	*
	Tap water	0.24 kg[35]	0.28 kg[36, 43]
Output	Polypropylene, granulate, recycled	0.83 kg	0.76 kg
	Municipal solid waste	0.17 kg[37]	0.24 kg[42, 44]

* As the same value as in the low impact.

Table S12. Life cycle inventory of Incineration (with energy recovery) among six plastic waste types and 18 research countries with lower and upper ranges.

Linked product name	linked activity name	LCI source	Low amount	Upper amount
Plastic waste of type i	treatment of waste plastic of type i, municipal incineration	Ecoinvent 3.8	1.00 kg	1.00 kg
Heat, district or industrial, natural gas	heat and power co-generation, natural gas, conventional power plant, 100MW electrical	Ecoinvent 3.8	-$E_{avoid,i,c,max}$*	-$E_{avoid,i,c,min}$*
Electricity, medium voltage	Market for electricity, medium voltage (country-specific)	Ecoinvent 3.8	-$E_{avoid,i,c,max}$*	-$E_{avoid,i,c,min}$*

* $E_{avoid,i,c,max}$ indicates the avoided net energy generation by incinerating 1 kg of plastic i in country c calculated by using the maximum value of net energy generation and the maximum value of the lower heating value of plastic in Eq.4. Ditto for $E_{avoid,i,c,min}$.

Table S13. Recovered electricity and heat from municipal waste incineration. Bottom ash for road construction and metal recycling are not considered. SYSAV is short for Sysav South Scania waste-to-energy plant.

Country	Region/ Name	Electricity production (GWh per year)	Heat production (GWh per year)	Waste Capacity (tonne per day)	LHV of waste (GJ/t)	Net heat production per tonne (GJ/t)	Net electricity production per tonne (kWh/t)
USA	(64 in total) [48]	13600	0	76712	10.6 [49]	0	486
Canada	Alexandria[50]	176	0	959	10.6 [49]	0	503
	Babylon[50]	136	0	671	10.6 [49]	0	555
	Burnaby[50]	192	0	770	10.6 [49]	0	683
	Emerald[51]	74.4	0	500	10.6 [49]	0	408
	Average	-	-	-	10.6	0	537
Mexico	Mexico City[52]	965	0	4384	13.3 [53]	0	603
Netherlands	(12 in total) [54]	-	-		10	2	528
Germany	Essen-Karnap[55]	333	1139	1918	-	5.9	476
	Hannover[56]	184	99	767	12.5	1.3	657
	Delfzijl[56]	191	8	1578	12	0.05	332
	Stapelfeld[56]	113	229	959	9.5	2.36	323
	Pirmasens[56]	98	38	493	11	0.76	544
	Helmstedt TRV[56]	308	0	1438	9.5	0	587
	Schwedt[56]	175	602	904	16.5	6.57	527
	Average	-	-	-	11.83	1.73	492
Austria	Spittelau[57]	60	500	685	-	7.2	240
	Zistersdorf[58]	106	0	445	10.5	0	750
	Average	-	-	-	10.5	3.6	495

Belgium	(14 in total)[59]	1500	350	7863	10.5	1.25	523
Spain	Sant Adrià de Besòs[60]	142	60	739	10.74[61]	0.8	526
France	Isséane[62]	75	699	1429	10.1	4.83	144
	Ivry-Paris XIII[63]	79	1047	1944	-	5.31	111
	Average	-	-	-	10.1	5.07	128
Italy	Acerra[64]	645	0	2006	10.7[65]	0	881
	Brescia[64]	600	900	2000	10.7[65]	4.44	822
	Corteolona[64]	51.62	0	171	10.7[65]	0	814
	Silla 2[64]	290	480	1479	10.7[65]	3.2	537
	Average	-	-	-	10.7	1.91	764
United Kingdom	Greatmoor[66]	29.4	6.6	822	12[67]	0.69	858
	Severnside[66]	37	10	959	12[67]	0.9	926
	EnviRecover[66]	20.4	3	548	12[67]	0.47	893
	Average	-	-	-	12	0.69	892
Poland	Poznan[68]	131	290	592	9	4.38	608
Taiwan, China	(24 in total)[69]	1950	0	24650	7.84[70]	0	217
Japan	Minato[71]	176	0	900	13.4	0	536
	Adachi[71]	130	0	700	12.1	0	507
	Chuo[71]	120	0	600	13.4	0	548
	Katsushika[71]	108	0	500	12.1	0	592
	Average	-	-	-	12.75	0	546
Turkey	Istanbul[72]	630	0	2740	7.5	0	630
Malaysia	Kajang[73]	71	0	1100	5.4	0	177
Indonesia	(12 in total)[74]	1872	0	16000	9.5[75]	0	351
Vietnam	Soc Son[76]	600	0	4000	7.5	0	411

Table S14. Comparison of lower heating values (LHV) reported in the literature for principal components of plastics in the waste stream (unit: MJ/kg). The table originated from Themelis, Castaldi, Bhatti and Arsova [77].

LHV, Plastic material	EIA, 2008	Stanford	Polymer Handbook	Franklin Associates	Phyllis database	Themelis, Castaldi, Bhatti and Arsova [77]
PET	20.5	23.2	-	24.7	21.9	23.9
HDPE	38	44.6	43.6	46.5	43.6	44.3
PVC	16.5	-	16.4	18.3	16.8	19.2
LDPE	24.1	42.2	-	46.2	43.5	44.3
PP	38	42.7	43.4	46.4	44.2	44.3
PS	35.6	42	39.2	-	44.2	41.5

Table S15. Substitution factors of secondary plastic to primary plastic regarding technical properties. The maximum and minimum values for each plastic are dropped when calculating the average value and selecting the range. The average range value of the substitution factor is assigned to plastic PS and PVC as no sufficient data is provided.

Quality ratio	HDPE	LDPE	PS	PVC	PET	PP	Technical property
Golkaram, Mehta, Taveau, Schwarz, Gankema, Urbanus, De Simon, Cakir-Benthem and van Harmelen [78]	-	0.92	-	-	-	0.87	Both mechanical and non-mechanical properties
Meys, Frick, Westhues, Sternberg, Klankermayer and Bardow [39]	0.7	0.7	0.9	-	1	0.7	Downcycling for polyolefins
Huysman, De Schaepmeester, Ragaert, Dewulf and De Meester [79]	0.72-0.94	0.72-0.94	-	-	-	-	Interfacial tension
Valentino [80]	-	-	-	-	0.9	-	Intrinsic viscosity [I]
Civancik-Uslu, Puig, Ferrer and Fullana-i-Palmer [81]	-	-	-	-	-	0.94	Flexural modulus [II] in Plastic sheet application
Gala, Raugei and Fullana-i-Palmer [82]	0.75	-	-	-	-	-	-
Demets, Van Kets, Huysveld, Dewulf, De Meester and Ragaert [83]	0.67	0.77	-	-	0.86	0.74	Elastic modulus [III]
Demets, Van Kets, Huysveld, Dewulf, De Meester and Ragaert [83]	1	0.81	-	-	0.77	0.92	Tensile strength [VI]
Demets, Van Kets, Huysveld, Dewulf, De Meester and Ragaert [83]	-	-	-	-	0.88	-	Intrinsic viscosity [V]
Average	0.82	0.81	0.82	0.82	0.82	0.83	-
Selected range	0.70-0.94	0.70-0.92	0.73-0.91	0.73-0.91	0.77-0.86	0.74-0.92	-

[I]: A measure of the duration of a dissolved polymer to dilute through a specified capillary. [II]: Flexural modulus denotes the ability of a plastic material to bend. [III]: The material's resistance to elastic deformation. [VI]: The material's ability to withstand an applied load without failure (tensile strength) or plastic deformation (yield stress).

3. Supplementary figure

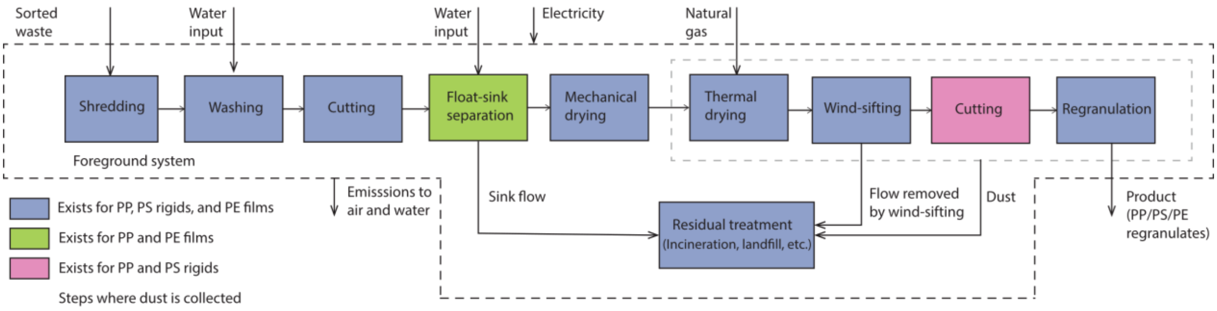

Fig. S1. The system boundary of mechanical recycling of waste PE, PS and PP in LCA. The figure is adapted from Civancik-Uslu, Nhu, Van Gorp, Kresovic, Larrain, Billen, Ragaert, De Meester, Dewulf and Huysveld [35]. We excluded the sorting and wastewater treatment processes.

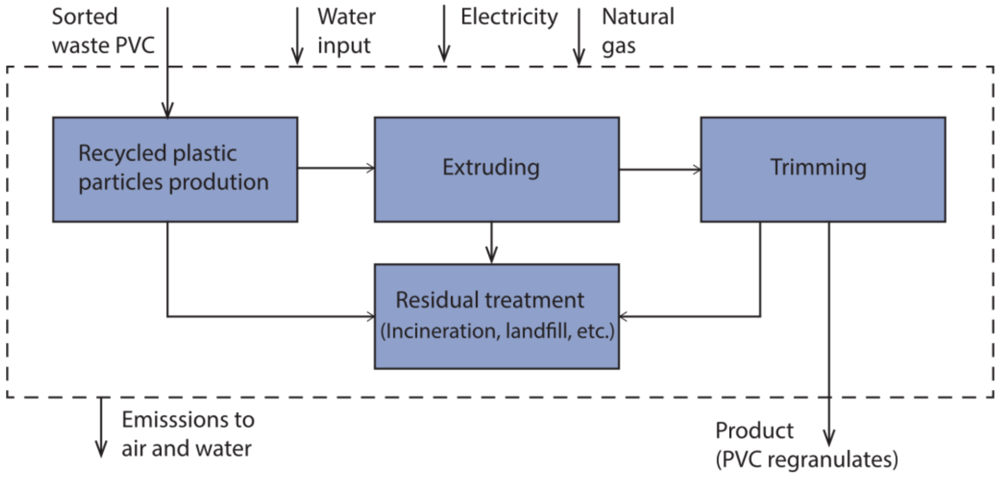

Fig. S2. The system boundary of mechanical recycling of waste PVC in LCA. The figure is adapted from Ye, Qi, Hong and Ma [45]. We excluded the collection and sorting processes.

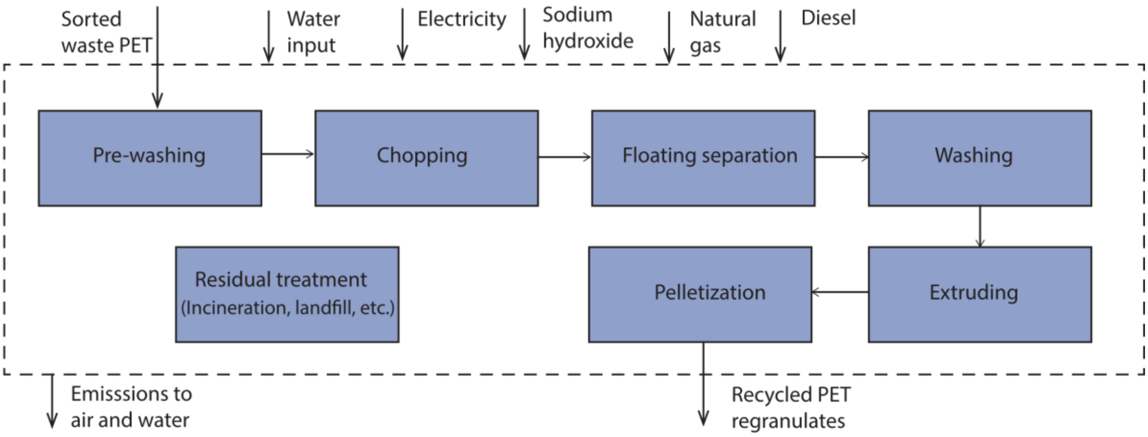

Fig. S3. The system boundary of mechanical recycling of waste PET in LCA. The figure is adapted from Shen, Worrell and Patel [84].

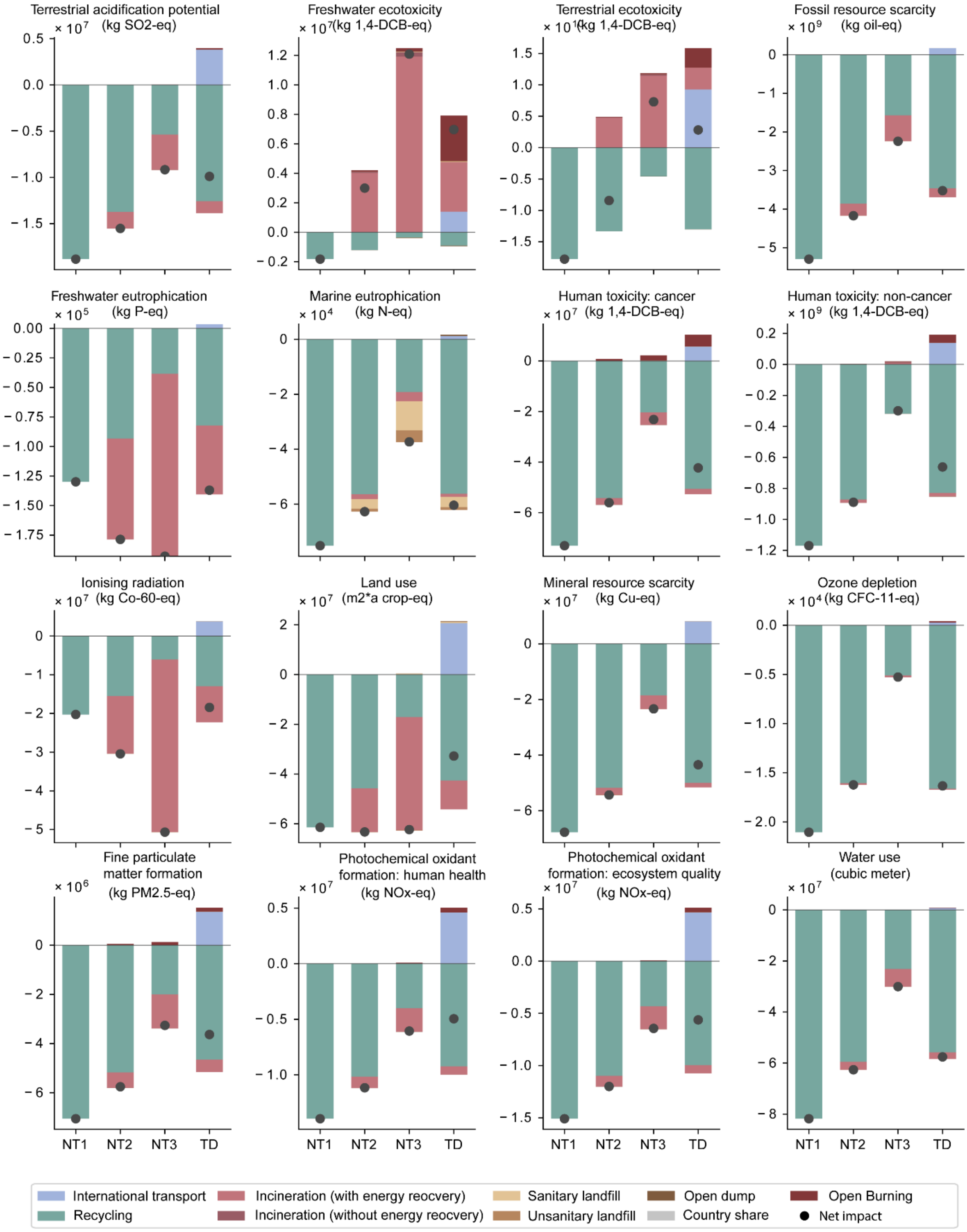

Fig. S4. The environmental impacts of plastic waste trade under four scenarios in 2022 across the remaining impact categories from the ReCiPe midpoint (H).

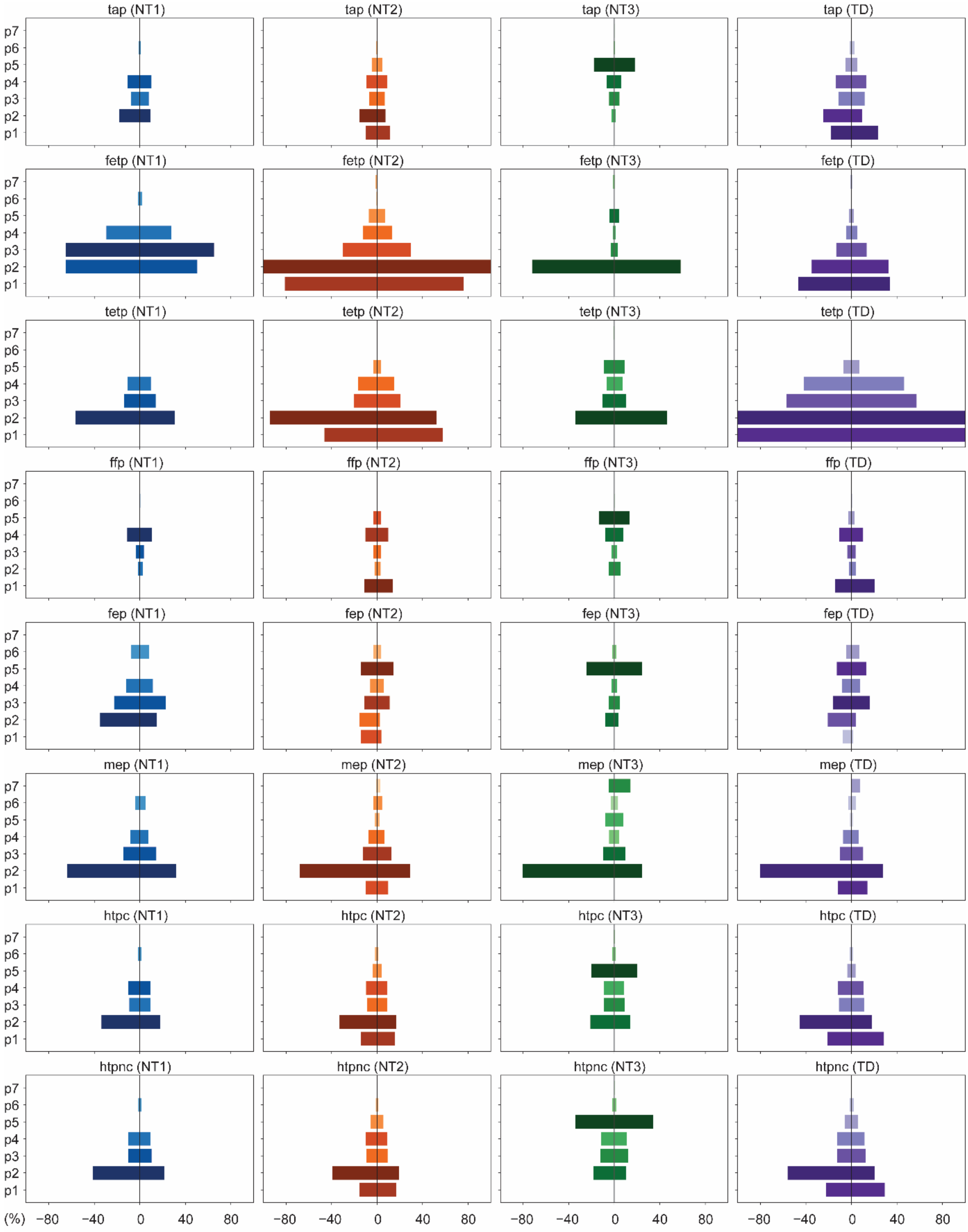

p1: Required recycling rate (RRR)
p2: Share of PET and PP in plastic waste 'Others'
p3: LCI of recycling
p4: Substitution factor of recycled platics
p5: LCI of inceration
p6: Share of HDPE and LDPE in plastic waste PE
p7: Treatment share between sanitary landfill, unsanitary landfill, and open dump

Fig. S5. Sensitivity analysis for the remaining impact categories under four scenarios (the first part). The variance exceeds or equals 100% as the bar reaches its endpoint. The length and

colour depth of the horizontal bars are proportional to the range of sensitivity results derived from pessimistic and optimistic cases. The characterization factors with full names are terrestrial acidification potential (tap; kg SO_2-eq), freshwater ecotoxicity (fetp; kg 1,4-DCB-Eq), terrestrial ecotoxicity (tetp; kg 1,4-DCB-Eq), fossil fuel potential (ffp; kg oil-eq), freshwater eutrophication potential (fep; kg P-eq), marine eutrophication potential (mep; kg N-eq), human toxicity potential (htpc; kg 1,4-DCB-eq), human toxicity potential (htpnc; kg 1,4-DCB-eq).

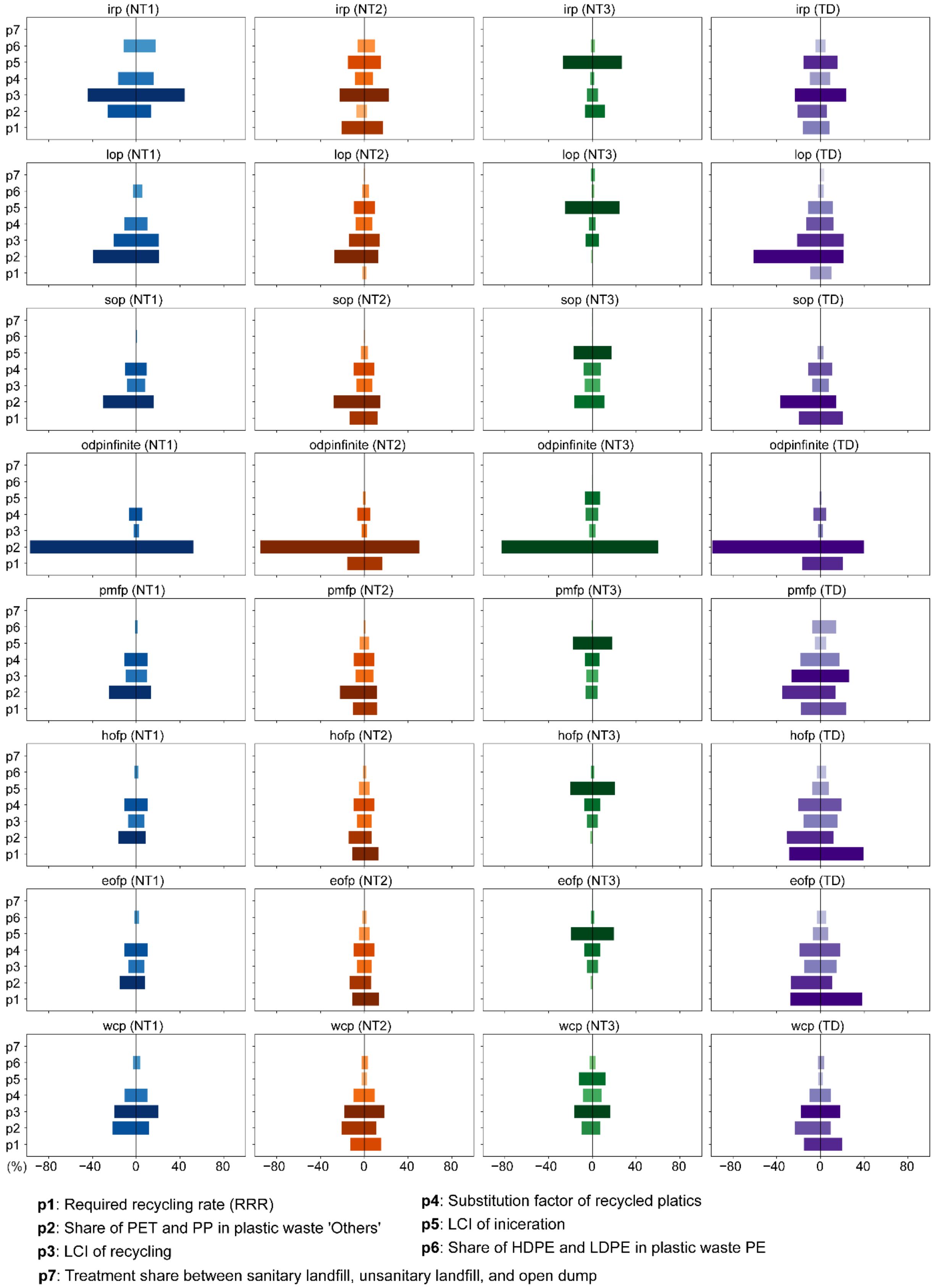

Fig. S6. Sensitivity analysis for the remaining impact categories under four scenarios (the second part). The variance exceeds or equals 100% as the bar reaches its endpoint. The length

and colour depth of the horizontal bars are proportional to the range of sensitivity results derived from pessimistic and optimistic cases. The characterization factors with full names are ionising radiation potential (irp; kg Co-60-eq), agricultural land occupation (lop; m2*a crop-eq), surplus ore potential (sop; kg Cu-eq), ozone depletion potential (odpinfinite: kg CFC-11-eq), particulate matter formation potential (pmfp; kg PM2.5-eq), photochemical oxidant formation potential: human health (hofp; kg NOx-eq), photochemical oxidant formation potential: terrestrial ecosystems (eofp; kg NOx-eq), water consumption (wcp; cubic meter).

4. References

(1) UN Comtrade. *UN Comtrade Database*. 2020. https://comtradeplus.un.org/ (accessed.

(2) Wen, Z.; Xie, Y.; Chen, M.; Dinga, C. D. China's plastic import ban increases prospects of environmental impact mitigation of plastic waste trade flow worldwide. *Nature Communications* **2021**, *12* (1), 425. DOI: 10.1038/s41467-020-20741-9.

(3) Bourtsalas, A. C.; Yepes, I. M.; Tian, Y. U.S. plastic waste exports: A state-by-state analysis pre- and post-China import ban. *Journal of Environmental Management* **2023**, *344*, 118604. DOI: https://doi.org/10.1016/j.jenvman.2023.118604.

(4) Liu, Z.; Liu, W.; Walker, T. R.; Adams, M.; Zhao, J. How does the global plastic waste trade contribute to environmental benefits: Implication for reductions of greenhouse gas emissions? *Journal of Environmental Management* **2021**, *287*, 112283. DOI: https://doi.org/10.1016/j.jenvman.2021.112283.

(5) Ren, Y.; Shi, L.; Bardow, A.; Geyer, R.; Suh, S. Life-cycle environmental implications of China's ban on post-consumer plastics import. *Resources, Conservation and Recycling* **2020**, *156*, 104699. DOI: https://doi.org/10.1016/j.resconrec.2020.104699.

(6) Sun, N.; Tabata, T. Environmental impact assessment of China's waste import ban policies: An empirical analysis of waste plastics importation from Japan. *Journal of Cleaner Production* **2021**, *329*, 129606. DOI: https://doi.org/10.1016/j.jclepro.2021.129606.

(7) Beyond Plastics and The Last Beach CleanUp. *The Real Truth About the U.S. Plastics Recycling Rate*; 2022. https://bit.ly/US-plastics-recycling-rate. United States Environmental Protection Agency. *Advancing Sustainable Materials Management: 2018 Tables and Figures*; 2020. https://www.epa.gov/sites/default/files/2021-01/documents/2018_tables_and_figures_dec_2020_fnl_508.pdf.

(8) Environment and Climate Change Canada. *Economic study of the Canadian plastic industry, markets and waste*; 2019. https://publications.gc.ca/site/eng/9.871296/publication.html.

(9) Statistics Canada. *Canada pilot physical flow account for plastic material 2019*. 2023. https://www150.statcan.gc.ca/n1/daily-quotidien/230309/dq230309e-eng.htm (accessed.

(10) Ambiente plastico. *Recycling rate in Mexico is 9.6%*. 2021. https://www.ambienteplastico.com/tasa-de-reciclaje-en-mexico-es-del-9-6/#:~:text=Expertos%20en%20residuos%20s%C3%B3lidos%20urbanos,sensibilizar%20sobre%20la%20correcta%20disposici%C3%B3n. (accessed.

(11) Ambiente plastico. *Recycling in Mexico: 6% of the plastic produced was reused in 2021*. 2022. https://www.ambienteplastico.com/reciclaje-en-mexico-6-del-plastico-producido-se-reutilizo-en-2021/ (accessed.

(12) Plastics Europe. *Plastics – the Facts 2020*; 2020. https://plasticseurope.org/wp-content/uploads/2021/09/Plastics_the_facts-WEB-2020_versionJun21_final.pdf.

(13) Plastics Europe. *The Circular Economy for Plastics – A European Overview*; 2022. https://plasticseurope.org/wp-content/uploads/2022/06/PlasticsEurope-CircularityReport-2022_2804-Light.pdf.

(14) Minister of Environment and Water (Malaysia). *Malaysia Plastics sustainability roadmap 2021-2030*; Malaysia, 2021. https://www.kasa.gov.my/resources/alam-sekitar/MALAYSIA-PLASTICS-SUSTAINABILITY-ROADMAP-2021-2030.pdf.

(15) World Economic Forum. *Radically Reducing Plastic Pollution in Indonesia: A Multistakeholder Action Plan National Plastic Action Partnership*; 2020. https://pacecircular.org/sites/default/files/2021-03/NPAP-Indonesia-Multistakeholder-Action-Plan_April-2020_compressed%20%281%29.pdf.

(16) Wasteforchange. *Surprising Recycling Facts*. 2019. https://waste4change.com/blog/fakta-daur-ulang-yang-mengejutkan/ (accessed.

(17) IUCN-EA-QUANTIS. *National Guidance for plastic pollution hotspotting and shaping action for Vietnam*. 2020. https://www.iucn.org/sites/default/files/content/documents/2021/vietnam_-_national_guidance_for_plastic_pollution_hotspotting_and_shaping_action.pdf (accessed.

(18) Vietnam communist review. *Plastic waste in Vietnam: Current situation and solutions*. 2022. https://www.tapchicongsan.org.vn/web/guest/bao-ve-moi-truong/-/2018/826009/rac-thai-nhua-o-viet-nam--thuc-trang-va-giai-phap.aspx (accessed.

(19) Environmental Protection Administration. *Solid Waste Statistics*; 2022. https://www.epa.gov.tw/eng/513B0B39D090DE4C.
(20) Japan Plastic Waste Management Institute. *An Introduction to Plastic Recycling in Japan 2019*; Tokyo, 2019. https://www.pwmi.or.jp/ei/plastic_recycling_2019.pdf.
(21) Japan Plastic Waste Management Institute. *Plastic Products, Plastic Waste and Resource Recovery 2019*; Tokyo, 2019. https://www.pwmi.or.jp/ei/siryo/ei/ei_pdf/ei50.pdf.
(22) Japan Plastic Waste Management Institute. *Plastic Products,Plastic Waste and Resource Recovery 2020*; Tokyo, 2020. https://www.pwmi.or.jp/ei/siryo/ei/ei_pdf/ei51.pdf.
(23) Japan Plastic Waste Management Institute. *An Introduction to Plastic Recycling in Japan 2022*; Tokyo, 2022. https://www.pwmi.or.jp/ei/plastic_recycling_2022.pdf.
(24) Anadolu Ajansı. *Türkiye recycles 1.1 million tons of plastic waste every year*. 2021. https://www.aa.com.tr/tr/cevre/turkiye-her-yil-1-1-milyon-ton-plastik-atigi-geri-donusturuyor/2427166# (accessed.
(25) Turkish Statistical Institute. *Waste Statistics 2020*. 2021. https://data.tuik.gov.tr/Bulten/Index?p=Atik-Istatistikleri-2021-37198 (accessed.
(26) Ekonomim. *The recycling rate in Turkey is only 30 percent*. 2023. https://www.ekonomim.com/yapi-kredi-ile-e-ticaret/turkiyede-geri-donusum-orani-sadece-yuzde-30-haberi-680902 (accessed.
(27) Chiong, M. S.; Chun, Y.-Y.; Tsukahara, K.; Tahara, K. An Analysis of Practices and Challenges for Plastic Recycling Industry in Malaysia. *International Journal of Automation Technology* **2022**, *16* (6). DOI: 10.20965/ijat.2022.p0831.
(28) The Circulate Initiative. *Mapping Local Plastic Recycling Supply Chains: Insights from Selected Cities in India, Indonesia, Thailand and Vietnam*. 2023. https://www.thecirculateinitiative.org/_files/ugd/77554d_9e849f45d51b4a2ebe01d12544f4a8cd.pdf?index=true (accessed.
(29) Lai, Y.-Y.; Lee, Y.-M. Management strategy of plastic wastes in Taiwan. *Sustainable Environment Research* **2022**, *32* (1), 11. DOI: 10.1186/s42834-022-00123-0.
(30) Drewniok, M. P.; Gao, Y.; Cullen, J. M.; Cabrera Serrenho, A. What to Do about Plastics? Lessons from a Study of United Kingdom Plastics Flows. *Environmental Science & Technology* **2023**, *57* (11), 4513-4521. DOI: 10.1021/acs.est.3c00263.
(31) Basuhi, R.; Moore, E.; Gregory, J.; Kirchain, R.; Gesing, A.; Olivetti, E. A. Environmental and economic implications of U.S. postconsumer plastic waste management. *Resources, Conservation and Recycling* **2021**, *167*, 105391. DOI: https://doi.org/10.1016/j.resconrec.2020.105391.
(32) Sevigné-Itoiz, E.; Gasol, C. M.; Rieradevall, J.; Gabarrell, X. Contribution of plastic waste recovery to greenhouse gas (GHG) savings in Spain. *Waste Management* **2015**, *46*, 557-567. DOI: https://doi.org/10.1016/j.wasman.2015.08.007.
(33) Picuno, C.; Alassali, A.; Chong, Z. K.; Kuchta, K. Flows of post-consumer plastic packaging in Germany: An MFA-aided case study. *Resources, Conservation and Recycling* **2021**, *169*, 105515. DOI: https://doi.org/10.1016/j.resconrec.2021.105515.
(34) Amadei, A.; Ardente, F. Modelling plastic flows in the European Union value chain. Publications Office of the European Union: Luxembourg, 2022.
(35) Civancik-Uslu, D.; Nhu, T. T.; Van Gorp, B.; Kresovic, U.; Larrain, M.; Billen, P.; Ragaert, K.; De Meester, S.; Dewulf, J.; Huysveld, S. Moving from linear to circular household plastic packaging in Belgium: Prospective life cycle assessment of mechanical and thermochemical recycling. *Resources, Conservation and Recycling* **2021**, *171*, 105633. DOI: https://doi.org/10.1016/j.resconrec.2021.105633.
(36) Lim, J.; Ahn, Y.; Kim, J. Optimal sorting and recycling of plastic waste as a renewable energy resource considering economic feasibility and environmental pollution. *Process Safety and Environmental Protection* **2023**, *169*, 685-696. DOI: https://doi.org/10.1016/j.psep.2022.11.027.
(37) Uekert, T.; Singh, A.; DesVeaux, J. S.; Ghosh, T.; Bhatt, A.; Yadav, G.; Afzal, S.; Walzberg, J.; Knauer, K. M.; Nicholson, S. R.; et al. Technical, Economic, and Environmental Comparison of Closed-Loop Recycling Technologies for Common Plastics. *ACS Sustainable Chemistry & Engineering* **2023**, *11* (3), 965-978. DOI: 10.1021/acssuschemeng.2c05497.
(38) Shan, C.; Pandyaswargo, A. H.; Onoda, H. Environmental Impact of Plastic Recycling in Terms of Energy Consumption: A Comparison of Mechanical and Chemical Recycling Technologies. *Energies* **2023**, *16* (5), 2199.

(39) Meys, R.; Frick, F.; Westhues, S.; Sternberg, A.; Klankermayer, J.; Bardow, A. Towards a circular economy for plastic packaging wastes – the environmental potential of chemical recycling. *Resources, Conservation and Recycling* **2020**, *162*, 105010. DOI: https://doi.org/10.1016/j.resconrec.2020.105010.
(40) Wernet, G.; Bauer, C.; Steubing, B.; Reinhard, J.; Moreno-Ruiz, E.; Weidema, B. The ecoinvent database version 3 (part I): overview and methodology. *The International Journal of Life Cycle Assessment* **2016**, *21* (9), 1218-1230. DOI: 10.1007/s11367-016-1087-8.
(41) USDA National Agricultural Library. *LCA Commons*. 2015. https://doi.org/10.15482/USDA.ADC/1173236 (accessed.
(42) Brouwer, M.; Picuno, C.; Thoden van Velzen, E. U.; Kuchta, K.; De Meester, S.; Ragaert, K. The impact of collection portfolio expansion on key performance indicators of the Dutch recycling system for Post-Consumer Plastic Packaging Waste, a comparison between 2014 and 2017. *Waste Management* **2019**, *100*, 112-121. DOI: https://doi.org/10.1016/j.wasman.2019.09.012.
(43) Larrain, M.; Van Passel, S.; Thomassen, G.; Van Gorp, B.; Nhu, T. T.; Huysveld, S.; Van Geem, K. M.; De Meester, S.; Billen, P. Techno-economic assessment of mechanical recycling of challenging post-consumer plastic packaging waste. *Resources, Conservation and Recycling* **2021**, *170*, 105607. DOI: https://doi.org/10.1016/j.resconrec.2021.105607.
(44) Arena, U.; Mastellone, M. L.; Perugini, F. Life Cycle assessment of a plastic packaging recycling system. *The International Journal of Life Cycle Assessment* **2003**, *8* (2), 92-98. DOI: 10.1007/BF02978432.
(45) Ye, L.; Qi, C.; Hong, J.; Ma, X. Life cycle assessment of polyvinyl chloride production and its recyclability in China. *Journal of Cleaner Production* **2017**, *142*, 2965-2972. DOI: https://doi.org/10.1016/j.jclepro.2016.10.171.
(46) Schwarz, A. E.; Ligthart, T. N.; Godoi Bizarro, D.; De Wild, P.; Vreugdenhil, B.; van Harmelen, T. Plastic recycling in a circular economy; determining environmental performance through an LCA matrix model approach. *Waste Management* **2021**, *121*, 331-342. DOI: https://doi.org/10.1016/j.wasman.2020.12.020.
(47) Plinke, E.; Wenk, N.; Wolff, G.; Castiglione, D.; Palmark, M. *Mechanical Recycling of PVC Wastes: Study for DG XI of the European Commission*; 2000. https://ec.europa.eu/environment/pdf/waste/studies/pvc/mech_recylce.pdf. Faraca, G.; Astrup, T. Plastic waste from recycling centres: Characterisation and evaluation of plastic recyclability. *Waste Management* **2019**, *95*, 388-398. DOI: https://doi.org/10.1016/j.wasman.2019.06.038.
(48) U.S. Energy Information Administration. *Biomass explained: Waste-to-energy (Municipal Solid Waste)*. 2022. https://www.eia.gov/energyexplained/biomass/waste-to-energy.php#:~:text=MSW%20is%20usually%20burned%20at,combustible%20MSW%20for%20electricity%20generation. (accessed 2022 17th November).
(49) Hitachi Zosen INOVA. *Olmsted / USA: Flexible Use of Energy through Sustainable Waste Management*. 2022. https://www.hz-inova.com/wiki/olmsted-usa/ (accessed 2022 21th November, 2022).
(50) COVANTA. *Our Facilities*. 2022. https://www.covanta.com/facilities (accessed 2022 17th November).
(51) Emerald Energy From Waste Inc. *Equipment*. 2022. https://emeraldefw.com/equipment/ (accessed 2022 17th November).
(52) Veolia group. *In Mexico City, Veolia will build and operate one of the largest waste to energy facilities in the world and the first in Latin America*. 2022. https://www.veolia.com/en/news/waste-to-energy-renewable-energy-mexico (accessed 2022 17th November).
(53) Escamilla-García, P. E.; Tavera-Cortés, M. E.; Pérez-Soto, F. Characterisation and calorific potential of waste generated in Mexico City for energy production. *International Journal of Environment and Waste Management* **2019**, *23* (2), 123-140. DOI: 10.1504/ijewm.2019.097611.
(54) M. de Leeuw; R. Koelemeijer. *Decarbonisation options for the Dutch waste incineration industry*; Netherlands Environmental Assessment Agency, 2022. https://www.pbl.nl/sites/default/files/downloads/pbl-2022-decarbonisation-options-for-the-dutch-waste-incineration-industry-4916.pdf.
(55) RWE. *Essen-Karnap waste-to-energy plant*. 2022. https://www.rwe.com/en/the-group/countries-and-locations/essen-karnap-waste-to-energy-plant (accessed 2022 14th November).

(56) EEW Energy from Waste. *Locations of EEW Energy from Waste GmbH*. 2022. https://www.eew-energyfromwaste.com/en/our-sites/ (accessed 2022 14th November).
(57) Wien Energie. *Spittelau waste incineration plant*. 2022. https://positionen.wienenergie.at/en/projects/spittelau-waste-incineration-plant/ (accessed 2022 10th November).
(58) FCC Environment. 2022. https://www.fcc-group.eu/files/documents/group/download/DL_letak_zisterdorf_EN_preview.pdf (accessed 2022 10th November).
(59) Belgian Waste-to-Energy. *Over BW2E*. 2022. http://www.bw2e.be/nl/over-bw2e/ (accessed 2022 15th November).
(60) TERSA. *Waste-to-Energy*. 2021. https://www.tersa.cat/en/valoritzacio-residus/valoritzacio-energetica/ (accessed 2022 15th November).
(61) Castrillón, L.; Fernández-Nava, Y.; González, A.; Marañón, E. A case study of the characteristics of municipal solid waste in Asturias (Spain): influence of season and source. *Waste Management & Research* **2013**, *31* (4), 428-431. DOI: 10.1177/0734242x12465463.
(62) Syctom. *Sorting/Transfer Centre and Energy Recovery unit in Isséane Annual Review 2021*; 2022. https://www.syctom-paris.fr/fileadmin/mediatheque/documentation/dip/DIP_2021_Isseane_01.pdf.
(63) Syctom. *Public Information Package 2021 IVRY – PARIS XIII*; Paris, 2022. https://www.suez.fr/-/media/suez-fr/files/ivry-paris-xiii/dip_2021.pdf?la=fr-fr.
(64) A2A Life Company. *Integrated waste cycle*. 2022. https://www.gruppoa2a.it/en/about-us/our-plants/waste-cycle (accessed 2022 13th November).
(65) Cucchiella, F.; D'Adamo, I.; Gastaldi, M. Municipal waste management and energy recovery in an Italian region. *Waste Management & Research* **2012**, *30* (12), 1290-1298. DOI: 10.1177/0734242x12462284.
(66) IDOM. *5 Waste to Energy Plants in Europe*. 2022. https://www.idom.com/en/project/wte-waste-to-energy-plants-in-ireland-poland-united-kingdom/ (accessed 2022 10th November).
(67) Tolvik Consulting. *UK Energy from Waste Statistics – 2021*; 2022. https://www.tolvik.com/wp-content/uploads/2022/05/Tolvik-UK-EfW-Statistics-2021_Published-May-2022.pdf.
(68) Hitachi Zosen INOVA. *Poland Energy-from-Waste Plant*; 2016. https://www.hz-inova.com/files/2016/10/201610_Poznan_E_Online.pdf.
(69) Environment Protection Administration. *4-6 The Operation of Municipal Waste Incineration Plants*; 2022. https://www.epa.gov.tw/DisplayFile.aspx?FileID=6545C8F049A6AB8B&P=acea9677-e6aa-4e9a-bb71-f62cc1e87d0f.
(70) Chen, Y.; Wang, C. Municipal solid waste (MSW) incineration's potential contribution to electricity production and economic revenue in Taiwan. *J. Taiwan Energy* **2017**, *4* (1), 93-106.
(71) Clean Authority of Tokyo. *List of incineration plants in 23 cities of Tokyo*. 2022. https://www.union.tokyo23-seisou.lg.jp.e.de.hp.transer.com/kojo/index.html (accessed 2022 17th November).
(72) Asian Infrastructure Investment Bank. *Türkiye: Istanbul Waste to Energy Generation Project*. 2021. https://www.aiib.org/en/projects/details/2021/approved/Turkey-Istanbul-Waste-to-Energy-Generation-Project.html (accessed 2022 15th November).
(73) Neville, A. Kajang Waste-to-Energy Plant, Semenyih, Malaysia. *Power* **2010**, *154* (12), 35-36.
(74) Reuters. *Indonesian president tells cities to build waste-to-energy plants*. 2022. https://www.reuters.com/article/us-indonesia-environment-energy-idUSKCN1UB1CG (accessed 2022 8th November).
(75) Qonitan, F. D.; Wayan Koko Suryawan, I.; Rahman, A. Overview of Municipal Solid Waste Generation and Energy Utilization Potential in Major Cities of Indonesia. *Journal of Physics: Conference Series* **2021**, *1858* (1), 012064. DOI: 10.1088/1742-6596/1858/1/012064.
(76) VNExpress. *Vietnam's largest waste-to-power plant to begin operation*. 2022. https://e.vnexpress.net/news/news/vietnam-s-largest-waste-to-energy-plant-begins-operation-4491922.html#:~:text=The%20VND7%20trillion%20(%24299.2,operation%20in%20the%20first%20phase. (accessed 2022 8th November).
(77) Themelis, N.; Castaldi, M.; Bhatti, J.; Arsova, L. Energy and economic value of nonrecycled plastics (NRP) and municipal solid wastes (MSW) that are currently landfilled in the fifty states. *Earth Engineering Center, Columbia University, New York* **2011**.

(78) Golkaram, M.; Mehta, R.; Taveau, M.; Schwarz, A.; Gankema, H.; Urbanus, J. H.; De Simon, L.; Cakir-Benthem, S.; van Harmelen, T. Quality model for recycled plastics (QMRP): An indicator for holistic and consistent quality assessment of recycled plastics using product functionality and material properties. *Journal of Cleaner Production* **2022**, *362*, 132311. DOI: https://doi.org/10.1016/j.jclepro.2022.132311.
(79) Huysman, S.; De Schaepmeester, J.; Ragaert, K.; Dewulf, J.; De Meester, S. Performance indicators for a circular economy: A case study on post-industrial plastic waste. *Resources, Conservation and Recycling* **2017**, *120*, 46-54. DOI: https://doi.org/10.1016/j.resconrec.2017.01.013.
(80) Valentino, G. Life Cycle Assessment of PET bottles: closed and open loop recycling in Denmark and Lombardy region. **2017**.
(81) Civancik-Uslu, D.; Puig, R.; Ferrer, L.; Fullana-i-Palmer, P. Influence of end-of-life allocation, credits and other methodological issues in LCA of compounds: An in-company circular economy case study on packaging. *Journal of Cleaner Production* **2019**, *212*, 925-940. DOI: https://doi.org/10.1016/j.jclepro.2018.12.076.
(82) Gala, A. B.; Raugei, M.; Fullana-i-Palmer, P. Introducing a new method for calculating the environmental credits of end-of-life material recovery in attributional LCA. *The International Journal of Life Cycle Assessment* **2015**, *20* (5), 645-654. DOI: 10.1007/s11367-015-0861-3.
(83) Demets, R.; Van Kets, K.; Huysveld, S.; Dewulf, J.; De Meester, S.; Ragaert, K. Addressing the complex challenge of understanding and quantifying substitutability for recycled plastics. *Resources, Conservation and Recycling* **2021**, *174*, 105826. DOI: https://doi.org/10.1016/j.resconrec.2021.105826.
(84) Shen, L.; Worrell, E.; Patel, M. K. Open-loop recycling: A LCA case study of PET bottle-to-fibre recycling. *Resources, Conservation and Recycling* **2010**, *55* (1), 34-52. DOI: https://doi.org/10.1016/j.resconrec.2010.06.014.
